# Characterisation of the unknown chemical composition of a commercial biodegradable agricultural plastic mulch film using complementary spectrometric and spectroscopic techniques†

**DOI:** 10.1039/d5an00423c

**Published:** 2025-07-03

**Authors:** Charlie Monkley, Michaela K. Reay, Helen L. Whelton, Richard P. Evershed, Charlotte E. M. Lloyd

**Affiliations:** a Organic Geochemistry Unit, School of Chemistry, University of Bristol Cantock's Close Bristol BS8 1TS UK cm17761@bristol.ac.uk charlotte.lloyd@bristol.ac.uk +44 (0)117 455 0991; b School of Geographical Sciences, University of Bristol University Road Bristol BS8 1SS UK

## Abstract

Biodegradable polyester mulch films are a viable alternative for use in agriculture to polyolefin-based films, offering reduced long-term microplastic pollution in agroecosytems with comparable protections for food security. However, these films carry diverse organic additives and non-intentionally added substances (NIASs), representing an underexplored source of anthropogenic chemicals in agroecosystems. Comprehensive chemical characterisation of these films is critical but hindered by restrictions on revealing proprietary formulations. This study presents a non-targeted screening (NTS) workflow employing multiple complementary analytical techniques to elucidate the organic composition of a polylactic acid (PLA)/polybutylene adipate-*co*-terephthalate (PBAT) mulch film. ^1^H nuclear magnetic resonance (NMR) quantified polyester contributions to the blend and revealed an unreported polybutylene sebacate (PBSe) component, likely from polybutylene sebacate terephthalate (PBSeT). Dissolution-precipitation extraction of the film followed by gas chromatography–mass spectrometry (GC-MS) and GC-flame ionisation detection (GC-FID) identified key additives in the extracted soluble fraction, including acetyl tributyl citrate (ATBC) plasticiser (4210 ± 135 μg g^−1^), and 8 cyclic oligoesters up to dimers. High-performance liquid chromatography-Orbitrap-mass spectrometry (HPLC-Orbitrap-MS) and direct infusion (DI)-Orbitrap-MS expanded oligoester detection to 83 additional components beyond the analytical window of GC-MS. The detailed oligoester profiles underscore the need to apply this workflow to biodegradable mulch films from diverse commercial sources and food industry applications to assess their broader chemical variability. These methodologies offer critical tools for the life cycle assessment of biodegradable agricultural plastic mulch films, advancing our understanding of the environmental impact and safety of these new materials.

## Abstract 2

The organic chemical composition of a biodegradable mulch film was determined using ^1^H NMR to quantify polyester composition, while GC-MS, DI-Orbitrap-MS and HPLC-Orbitrap-MS/MS identified >100 plastic-derived compounds, including >90 oligoesters.

## Graphical abstract

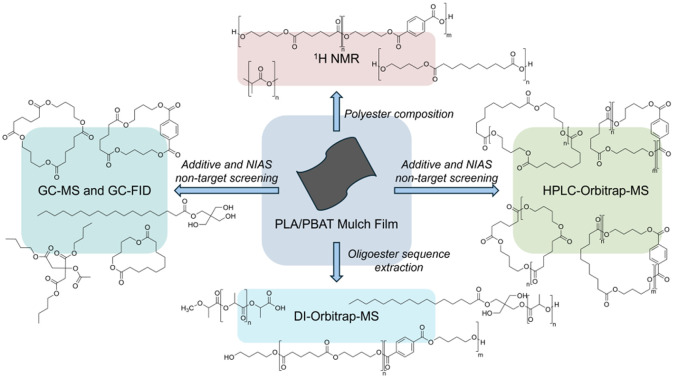

## Introduction

Agricultural plastic mulch film is one of the most economically viable and accessible cultivation practices, whereby, film is stretched over the soil prior to cropping to maintain a humid microclimate at the crop root-soil zone.^1^ Application of the film over the cropping season simultaneously acts as a protective barrier against pests, weeds and detrimental weather, leading to increased water use efficiency and crop yields.^2–4^ China is the predominant country to adopt agricultural plastic mulching practices and in 2012, it is estimated to have increased the production of wheat, maize, and rice by 30 million tonnes.^5^ Agricultural plastic mulching also reduced the use of irrigation water by 35 million m^3^ per year and herbicide use by 16 000 tonnes per year in China, helping to provide food for approximately 85 million people annually.^5^ Thanks to these benefits, agricultural plastic much film aids food security, especially for those in resource-constrained regions or adverse and changing climates.^6^

However, the use of low density polyolefin-based mulch films, such as polyethylene (LDPE) or polypropylene (PP), brings with them potential negative environmental impacts.^7^ These arise from the accumulation of long-lived macro- (>5 mm) and/or microplastic (<5 mm) particles in agroecosystems as a consequence of improper disposal or fragmentation.^8,9^ Because of this, biodegradable mulches have been developed as a means of alleviating long-term agroecosystem plastic pollution, comprising polyesters, including polybutylene-*co*-adipate terephthalate (PBAT), poly(butylene succinate) (PBS) and poly(ε-caprolactone) (PCL) often blended with compostable polylactic acid (PLA) or naturally occurring polysaccharides.^10^ Such films have been shown to have comparable agronomic benefits to LDPE films for multiple crop types.^11,12^

In addition to the polymer matrix of plastics, inorganic and organic chemical additives are incorporated to aid with processing, improve functionality, alter aesthetics and reduce production costs.^13–15^ Both additives and polymers are subject to degradation, transformation or intermediate formation during production and infield use, to non-intentionally added substances (NIASs). There is potential for these diverse chemical components to be released to soils used for food production through leaching from the bulk material or following material degradation in cases where the biodegradable mulch film is unrecovered or tilled into the soil after functional use.^16^ Therefore, for biodegradable mulches to be a successful alternative to polyolefin films, the additive content, along with the bulk polymeric composition and any derivatives it may form, must be passively incorporated into the environment without negative implications to ecosystem functioning.^17^ A recent EU standard for biodegradable mulches intended for end-of-use tilling recognises this, mandating (eco)toxicity assessments (for plants, worms and nitrifying bacteria) for the material as a whole and for chemical constituents incorporated above 1%, whilst also limiting compounds listed by the European Chemicals Agency (ECHA) as substances of very high concern (SVHC) to specified thresholds.^18,19^

Comprehensive characterisation methodologies are essential for life cycle assessment of biodegradable mulch films.^20^ The polymer composition is usually reported by manufacturers and can be confirmed with spectroscopic or spectrometric techniques either for pristine or aged material. For instance, Nelson *et al.*^21^ demonstrated the utility of ^1^H nuclear magnetic resonance (NMR) spectroscopy in identifying and quantifying residual PLA/PBAT mulch films in soils and it has further application for the monitoring of PLA/PBAT biodegradation.^22,23^ In comparison, Fourier transform infrared (FTIR) spectroscopy suffers from poorer spectral resolution to NMR and lacks quantitative precision required for mixed polyester plastics, but still holds value for monitoring functional group changes during biodegradation.^22,23^ Alternatively, pyrolysis-gas chromatography-mass spectrometry (Py-GC-MS) may be combined with thermogravimetric analysis (TGA) to provide quantitative polymer composition,^24^ and multi-shot Py-GC-MS can also characterise additives *via* initial thermal desorption.^25^ Overall, polyester compositional analysis using ^1^H NMR is the preferred technique for detailed characterisation of PLA/PBAT-based films, owing to the insight it provides into molecular structure, including monomer identity, ratio, and sequence connectivity. Crucially, NMR analysis is made feasible by the high solubility of both PBAT and PLA in deuterated chloroform (CDCl_3_), which allows for effective dissolution and reliable spectral acquisition.^26^

Beyond the main polymer components, the additive components of mulch films are rarely specified as they are regarded as propriety information. For additive component characterisation of mulch films, targeted analyses are typically used for additives of known environmental or human health concern, which involves solvent extraction followed by hyphenated mass spectrometry (MS) analyses. For example, reverse-phase high performance liquid chromatography (HPLC) coupled with electrospray ionisation mass spectrometry (ESI-MS) has been effective for benzotriazoles, benzophenones, and hindered amine light stabilisers (HALS),^27,28^ and organophosphite/organophosphate antioxidants.^29^ Additionally, GC-MS and GC-high resolution MS (GC-HRMS) is commonly used for determination of phthalate and adipate plasticisers.^30–32^ Therefore, to obtain a complete perspective of the chemical composition of plastic mulch films, a range of analytical techniques are required.

Importantly, the chemical complexity of biodegradable mulch films extends beyond additive components typically evaluated as substances of known concern. Non-targeted screening (NTS) of PLA/PBAT mulch films by Cui *et al.*^33^ detected 80 components *via* GC-MS, including 23 alkane lubricants, 9 amide slip agents and 5 fatty acids, which do not present any environmental hazards, whereas other additives of concern were identified including 3 phthalates and bisphenol A, which are known to cause reproductive toxicity.^19^ Xu *et al.*^30^ analysed PLA/PBAT mulch film extracts using GC-quadrupole time-of-flight (QTOF)-MS and LC-QTOF-MS but only reported 45 additive components, which included priority phthalates, bisphenols and organophosphate triesters.^19^ NTS reduces detection bias compared to targeted approaches but other organic components of PLA/PBAT mulches remain unexplored. Polyester synthesis generates oligoester by-products, which can comprise significant portions of pristine materials.^34–37^ Reay *et al.*^16^ found PLA/PBAT derived NIASs to comprise 37.4% of the detected organic content, which was greater than the combined additive content (24.3%). Biodegradation further increases oligoester derivatives,^38^ and concern has been raised due to the potential for polyester-derived chemicals to interfere with microbial functioning or alter soil properties.^39^ While the aforementioned reports have provided important insight into the potentially complex compositions of biodegradable mulch films, they do not provide a workflow which allows chemical composition (bulk polymer, intentional and unintentional chemical components) of commercially available mulch films to be defined for use in environmental risk assessments.

Herein, we present a NTS workflow designed to characterise the comprehensive composition of organic components in PLA/PBAT mulch films. Sample preparation strategies and a combination of complementary analytical techniques are presented that detect chemical components across the molecular weight range, from volatiles to polymers. Bulk polyester composition was characterised with ^1^H NMR, and hyphenated chromatography MS techniques utilised for the characterisation of solvent extracted components. The dissolution-precipitation extraction procedure was chosen due to its universal applicability and assurance of dissolving the polyester matrix,^26^ ensuring additive and NIAS recovery to the extraction solvent was not hindered by limited diffusion through the polymer matrix.^13^ A key novelty lies in the tailored MS data analysis workflows, which overcome challenges posed by unknown composition. Overall, this approach enables the detection and identification of both additives and NIASs, as well as unreported polymer components, thereby fully addressing the chemical complexity of these materials, which would be of value in supporting environmental life cycle assessments.

## Experimental

### Chemicals, reagents and sample collection

Information on solvents, plastic, additive and oligoester standards is presented in the ESI (Section S1).† The internal standards benzyl benzoate (CAS 120-51-4, ≥99% purity, 2.01 mg mL^−1^) and butyl benzyl phthalate (BBP) (85-68-7, >98%, 1.98 mg mL^−1^), and *N*-methyl-*N*-(trimethylsilyl)trifluoroacetamide (MSTFA) for derivatisation, were purchased from Sigma-Aldrich, Merck Life Sciences UK Ltd.

A commercially available, biodegradable mulch film of reported composition 85% PBAT and 15% PLA (PLA/PBAT mulch) was purchased directly from a UK manufacturer. The pristine PLA/PBAT mulch was cut to ∼5 mm^2^ pieces and stored in furnaced aluminium foil prior to extraction and analysis.

### Polymer compositional analysis: ^1^H NMR

^1^H NMR was used to confirm the polymeric composition of the mulch film and their relative proportions. Plastic mulch and polymer standards (PLA, PBAT and PBSe) (5 mg) were dissolved in 700 μL deuterated chloroform (CDCl_3_) within Norell® Select Series™ 5 mm NMR tubes (NORS55007).

^1^H NMR spectra were recorded on a 600 MHz Bruker Neo spectrometer equipped with a 5 mm TXO cryogenically-cooled probe at 25 °C. Topspin 4.1.3 was used for data acquisition and processing. The standard Bruker PROTON experiment acquisition parameters were used and the analysis software was MestReNova 14.2.1.

Molar ratios were determined through the following equation, using assigned proton signals that represent the monomeric units of each polymer:^40^
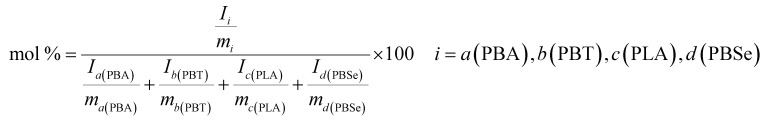
where *I* is the intensity of the assigned ^1^H NMR signal for the repeated monomeric unit of the polymer, *m* is the number of protons in the assigned ^1^H NMR signal for the monomeric unit of the polymer, *a*(PBA), *b*(PBT), *c*(PLA) and *d*(PBSe) are ^1^H NMR signals for PBA, PBT, PLA and PBSe moieties, respectively.

By using the same equation but multiplying the molar equivalents by the molecular weight of the repeat units, the weight percentage of the polymers were calculated, assuming that the contribution from end groups is negligible. Additional additive mass is not accounted for.
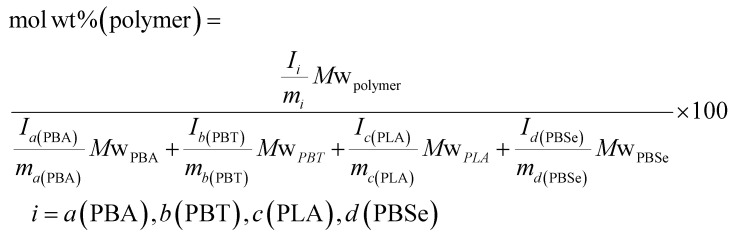
where *M*w_PBA_, *M*w_PBT_, *M*w_PLA_ and *M*w_PBSe_ are the molecular weights of the monomeric units of PBA, PBT, PLA and PBSe. *M*w_polymer_ refers to the polymer of interest of the three.

### Dissolution-precipitation extraction procedure

**Fig. 1:**
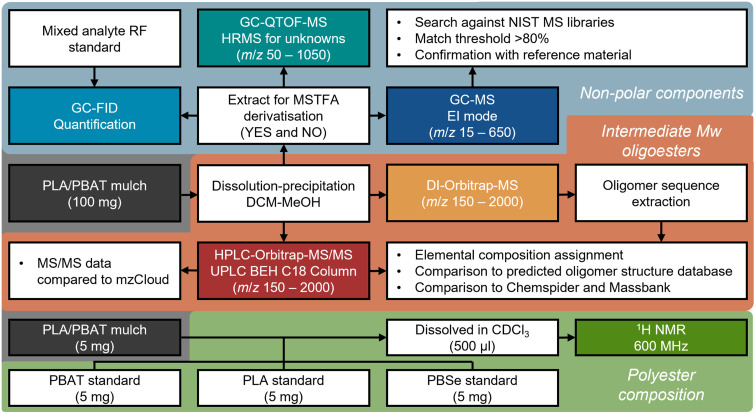
Overview of dissolution-precipitation extraction procedure and subsequent analytical workflow with instrumental analyses highlighted in coloured cells.

An overview of the extraction procedures, recovery assessment and characterisation considerations are outlined in Fig. 1. Extracts and procedural blanks were produced in triplicate. PLA/PBAT mulch (0.1 g) was dissolved in 2 mL DCM and 30 μL of 2.01 mg mL^−1^ benzyl benzoate was added as an internal standard (IS). Extracts were sonicated (10 min) before polymer was precipitated with the gradual addition of MeOH (10 mL) and vortex mixed (30 s). Extracts were filtered through pre-combusted 1.2 μm glass microfibre filters (Whatman® GF/C Grade; Merck Life Sciences Ltd), the remaining polymer was washed with MeOH (2 × 5 mL) and the combined extracts were evaporated under N_2_ to <2 mL at room temperature. At this point, polymer was observed to evolve from solution as suspended “white precipitate”. Extracts were centrifuged at 3000 rpm (3 min) and supernatant was transferred. This was repeated three times with 1 mL MeOH and the supernatants combined. The precipitate was dried at 50 °C and confirmed as residual polymer (Section S2; ESI†). MeOH extracts were evaporated to dryness under N_2_ and redissolved in 250 μL of MeOH.

Extracts were analysed both with and without MSTFA derivatisation for GC-FID and GC-MS. For derivatisation, a dried sub-sample (50 μL) was derivatised with MSTFA (30 μL, 1 h, 70 °C). Excess MSTFA was evaporated under N_2_, and the derivatised extract was diluted in ethyl acetate (GC-FID and GC-MS, this was 1 : 10 by redissolving in 500 μL and for GC-QTOF-MS, this was 1 : 50).

### GC-FID analyses

GC analysis for quantification was undertaken on a Thermo Fisher Scientific™ Trace™ 1300 Gas Chromatograph fitted with a non-polar Agilent HP-1 column (50 m × 0.32 mm, 0.17 μm film thickness). Extract (1 μL) was introduced to the programmable temperature vaporiser (PTV) inlet at an injection temperature of 50 °C in splitless mode for 2 min, followed by split flow at 12 mL min^−1^. The oven temperature programme was: 50 °C (1 min), ramping (5 °C min^−1^) to a final temperature of 300 °C (10 min). Helium (He) carrier gas was set at a constant flow rate of 2 mL min^−1^. The flame ionisation detector (FID) was held at 320 °C with a data collection rate of 10 Hz. Data collection and analysis was performed in Chromeleon® 7 (Version 7.2.1.5833; Thermo Fisher Scientific™).

### GC-MS analyses

GC-MS analysis for compound identification was undertaken on a Thermo Fisher Scientific™ ISQ™ LT single quadrupole GC-MS system. The GC set up was the same as used in GC-FID analyses. The MS operated under electron ionisation (EI) (70 eV) under a full scan range (*m*/*z* 15–650) at a scan time of 0.2 s with a scan delay of 5 min. The transfer-line to the MS and ion source were maintained at 300 °C, and the carrier gas (He) was set at a constant flow rate of 2 mL min^−1^.

### Analyte identification, quantification and recovery assessment with GC-FID and GC-MS

GC-MS data analysis was conducted using Xcalibur Version 4.1.31.9 (Thermo Fisher Scientific™ Ltd). Spectral libraries included: NIST 14 Main Library, NIST 2019 Additives Library, NIST 2019 Flame Retardants Library, NIST Stabilisers-Antioxidants Library and the NIST 2019 Plasticisers Library. Compounds with structural matches >80% were tentatively identified in extracts and those identified were confirmed with reference to standards, where available. Quantification of each analyte and recovery of the IS was with reference to the amount of known BBP solution (125 μL 1.98 mg mL^−1^; 250 μg), added post extraction. Response factors (RF) were determined with reference compounds of suspects (0.10 mg mL^−1^) relative to BBP using GC-FID. When a reference compound was unavailable, the RF for a chemically similar surrogate was used for quantification. The relative recovery for analytes with available reference material is outlined in the ESI (Section S3).† Additionally, GC-QTOF-MS was used for the structural elucidation of unknown PLA/PBAT mulch film components not present in MS libraries (Section S4; ESI).†

### DI-Orbitrap-MS analyses

Direct infusion (DI)-Orbitrap-MS was utilised for the detection of oligomeric components in extracts. Method development with 1,6,13,18-tetraoxacyclotetracosane-7,12,19,24-tetraone, pharacine and cyclotris(1,4-butylene terephthalate) (10 μg mL^−1^ in MeOH) found optimal ionisation using a mobile phase of MeOH with 0.5 mM ammonium acetate for the formation of [M + NH_4_]^+^. No analyte ion signals distinct to background ion signals or solvent blanks were found in negative mode (spray voltage −2500 V). Dissolution precipitation extracts and procedural blanks were diluted by 1 : 1000 in MeOH, equivalent to a final BBP concentration of 1 μg mL^−1^. The Orbitrap-MS was calibrated with Pierce™ FlexMix™ Calibration Solution (Thermo Fisher Scientific) prior to analyses and MeOH blanks (10 μL) were injected between each sample.

DI-Orbitrap-MS was undertaken using the HPLC stack for consistent flow rates and injection volumes while bypassing the column. The system comprised a Thermo Scientific™ UltiMate™ 3000 UHPLC System coupled with a Thermo Scientific™ Orbitrap ID-X mass spectrometer. The mobile phase was MeOH (0.5 mM ammonium acetate) which was held at a constant flow rate of 0.2 mL min^−1^. The extract (10 μL) was injected and flowed directly to the fitted heated-ESI (HESI) probe. The ion source was set to positive polarity mode with a spray voltage of +3500 V. Sheath gas (25 arbitrary units (arb)), auxiliary gas (10 arb) and sweep gas (4 arb) were maintained along with constant ion transfer tube temperature (300 °C) and vaporiser temperature (50 °C). Total acquisition time was 6 min with Orbitrap resolution set to 240 000 in profile mode with an *m*/*z* scan range of 150–2000.

Thermo .raw files were centroided and outputted as .mzML files using the MSConvertGUI software for analysis,^41^ which was adapted from the work of Pemberton^42^ and detailed in the ESI (Section S5).†^43,44^ In brief, ion signals separated by a constant *m*/*z* difference equivalent to the monomeric units for PLA (72.02113 Da), PBA (200.10486 Da), PBT (220.07356 Da) and PBSe (256.16746 Da) were extracted as oligomer sequences within *R*.^45^ This was constrained at an intensity threshold of 1 × 10^4^ and within a mass tolerance of ±0.001 Da. Following sequence construction, each ion was assigned an elemental composition, which additionally informed the adduct formed (*e.g.* [M + NH_4_]^+^ or [M + Na]^+^) along with any isotope contribution (*e.g.*^13^C or ^18^O) (isotopologues could be disregarded).

The elemental composition of the first ion in a sequence was compared to a compiled predicted structure database, which included 91 linear and cyclic combinations of the monomer components from each polyester component in the system (ESI; Spreadsheet 1).† Tentative structural assignment for the lowest ion in the series facilitated analogous assignment of consecutive ions, which would increase by a single monomer unit each time. In cases where comparison to the compiled predicted structure database was ineffective, molecular formula were compared to the ChemSpider online databases and interpreted for probable chemical components based on those already characterised through GC-MS and GC-QTOF-MS. Accurate molecular formula assignment along with tentative structural information sets identification confidence for oligoester components detected through this approach at level 3, of 5, on the scale set by Schymanski *et al.*^46^

### HPLC-Orbitrap-MS analyses

Dissolution precipitation extracts and procedural blanks were diluted by 1 : 100 into MeOH (BBP concentration of 10 μg mL^−1^) for reverse phase HPLC-Orbitrap-MS. A mixed solution of reference material that included 1,6-dioxacyclododecane-7,12-dione, pharacine, BBP, ATBC, cyclotris(1,4-butylene terephthalate) and pentaerythritol monostearate (PMS) was prepared in MeOH at 10 μg mL^−1^ and monitored over the analytical run for consistency in retention times (RTs) (maximum deviation: ±0.04 min) and mass accuracy (maximum RSD of precursor ion: 7 × 10^–5^%).

The HPLC-Orbitrap-MS system comprised the same HPLC stack and MS as that used for DI-Orbitrap-MS, with the addition of an Acquity C18 BEH column (2.1 × 150 mm, 1.7 μm particle size), which was maintained at 45 °C. The mobile phase consisted of: (A) H_2_O and (B) MeOH, both with 0.5 mM ammonium acetate. The same solvent system with the addition of 0.1% formic acid was tested but did not result in the detection of novel compounds or present any advantage compared to 0.5 mM ammonium acetate alone. The solvent gradient started at 50% (B) for 3 min, which was then linearly ramped to 95% (B) at 24 min, held at 95% (B) for 20 min, and returned to 50% (B) by 46 min for 10 min equilibration.

The same source and MS scan properties were as outlined for DI-Orbitrap-MS analysis were used. Alongside positive MS data acquisition, data-dependent MS/MS (*dd*MS^2^) occurred with the number of data-dependent scans set to 5 and isolation of precursor ions within an isolation of *m*/*z* 1 using the quadrupole trap. Fragmentation was achieved with stepped higher-energy collisional dissociation (HCD) energies at 20, 30 and 50% of the instrument's maximum. A dynamic exclusion window of 10 s was set with a mass tolerance of 3 ppm to limit repeat ion fragmentation, and an intensity threshold was set based on the signal intensity of the baseline (1 × 10^6^).

For analyte characterisation, peak by peak analysis was undertaken within Xcalibur for the assignment of precursor ions subjected to *dd*MS^2^. Elemental composition, adduct formation and molecular formula were assigned in all cases. Molecular formula could then be compared to online spectral databases (ChemSpider, MassBank) for an initial list of suspect hits, and to the constructed database of expected oligoester components. MS/MS data was additionally searched against the mzCloud HRMS library for matches in both precursor ion accurate mass and corresponding fragment ions. In cases where cyclic oligoester components were absent from spectral libraries, diagnostic fragment ions provided additional structural information to determine the monomeric components, previously assigned in Monkley *et al.*^37^ Therefore, the confidence of identification fits the requirements for level 2 set by Schymanski *et al.*^46^ for probable structures in all cases. This was improved to confirmed structures with reference material where available (Table 1).

**Table 1:** Overview of detected PLA/PBAT mulch organic components with GC-MS, DI-Orbitrap-MS (oligomer sequence extraction) and HPLC-Orbitrap-MS/MS

Assignment	Type	*n*	*m*	GC-MS characteristic ions/*m*/*z*	DI-Orbitrap-MS	HPLC-Orbitrap-MS/MS characteristic ions/*m*/*z*	Confirmed structure	Concentration/μg g^−1^
ATBC	Plasticiser	–	–	185, 129, 259	ND	129.01831, 185.08102, 139.00272	✓	4210 ± 135
TBA	NIAS	–	–	113, 157, 57	ND	139.00261, 157.01319, 111.00773		*1400* ± *170*
TBC	Plasticiser	–	–	185, 129, 57	ND	ND		*104* ± *23*
Palmitic acid*	Lubricant	–	–	117, 313, 129	ND	ND	✓	*18* ± *3*
Stearic acid*	Lubricant	–	–	117, 129, 132	ND	ND	✓	*4* ± *2*
PMP*	*Unknown*	–	–	191, 244, 73	✓	357.29994, 71.04916, 101.05974		
PMP-LA*	*Unknown*	–	–	191, 244, 147	✓	429.32104, 83.04915, 95.08555		
PMP-[LA]_*n*_	*Unknown*	2–4	–	ND	✓	ND		
PMS*	*Unknown*	–	–	117, 191, 73	✓	385.33136, 71.04916, 83.04916	✓	
PMS-LA*	*Unknown*	–	–	117, 191, 73	✓	457.35227, 83.04912, 95.08551		
PMS-[LA]_*n*_	*Unknown*	2–6	–	ND	✓	ND		
[AA-BD]	Cyclic/NIAS	–	–	55, 129, 111	✓	ND	✓	168 ± 32
[AA-BD]_2_	Cyclic/NIAS	–	–	129, 111, 201	✓	201.11221, 111.04412, 401.21721	✓	772 ± 5
[AA-BD]_*n*_	Cyclic/NIAS	3–5	–	ND	✓	201.11221, 111.04412, 401.21721		
[AA-BD]_6_	Cyclic/NIAS	–	–	ND	✓	ND		
[AA-BD]-[TA-BD]	Cyclic/NIAS	–	–	149, 104, 221	✓	221.08086, 149.02332, 111.04408		*480* ± *12*
[AA-BD]_*m*_-[TA-BD]_*n*_	Cyclic/NIAS	2–3	1	ND	✓	387.10743, 221.08101, 149.02334		
		1–3	2–4	ND	✓	221.08084, 421.18570, 149.02333		
		1–4	5	ND	*n* = 1–3	221.08083, 387.10739, 421.18582		
		1–3	6	ND	*n* = 1	201.11219, 221.08089, 401.21704		
		1	7	ND	–	201.11222, 221.08093, 401.21714		
[AA-BD]_*n*_-[TA-BD]	Linear/NIAS	2–4	–	ND	✓	ND		
BD-[AA-BD]_*n*_	Linear/NIAS	2–4	–	ND	✓	ND		
BD-[AA-BD]_*m*_-[TA-BD]_*n*_	Linear/NIAS	1,2	1	ND	✓	ND		
		2,3	2	ND	✓	ND		
		1, 3	3	ND	✓	ND		
		1–2	4	ND	✓	ND		
Me-[LA]_7_-[AA-BD]_*n*_	Linear/NIAS	0–2, 4	–	ND	✓	ND		
[TA-BD]_2_	Cyclic/NIAS	–	–	132, 149, 104	✓	387.10762, 149.02344, 369.09713	✓	348 ± 9
[TA-BD]_3_	Cyclic/NIAS	–	–	ND	✓	149.02336, 387.10745, 589.17051	✓	
[TA-BD]-[SeA-BD]	Cyclic/NIAS	–	–	149, 221, 166	✓	149.02332, 405.19086, 221.08094		*297* ± *7*
[TA-BD]_*m*_-[SeA-BD]_*n*_	Cyclic/NIAS	2–5	1	ND	*n* = 2–4	149.02336, 203.12774, 257.17491		
		1–4	2	ND	✓	149.02336, 203.12774, 387.10728		
		1–4	3	ND	*n* = 1–3	149.02336, 387.10728, 203.12774		
[SeA-BD]	Cyclic/NIAS	–	–	166, 98, 138	✓	185.11730, 257.17473, 203.12781		*588* ± *15*
[SeA-BD]_2_	Cyclic/NIAS	–	–	185, 257, 166	✓	185.11730, 257.17491, 513.34218		*252* ± *3*
[SeA-BD]_*n*_	Cyclic/NIAS	3–4	–	ND	✓	257.17487, 203.12789, 185.11733		
[LA]_2_	Cyclic/NIAS	–	–	56, 28, 43	–	ND	✓	
[LA]_*n*_	Cyclic/NIAS	6–12		ND	✓	ND		
[LA]_*n*_-Me	Linear/NIAS	2	–	ND	✓	ND		
		3–10	–	ND	✓	105.05464, 145.04955, 89.05973		
		11–20	–	ND	✓	ND		

### Quality assurance considerations

Stringent control measures are required to limit potential additive contamination following leaching from plastic equipment. Aluminium foil and glass were used for storage, extraction vessels or other practical equipment where possible, and cotton lab coats were worn (Fisher Scientific™ Ltd). All glassware, glass fibre filters and aluminium foil was wrapped in aluminium foil and furnaced at 450 °C for 2 h prior to use. Volumetric equipment was cleaned by successive washes with double distilled water and solvents of different polarity (*n*-hexane, MeOH and DCM) before drying. Handling of filters and films was done with solvent sterilised tweezers. Effort was made to minimise sample exposure time to the open air by sealing vials with furnaced stoppers or covering with aluminium foil. Procedural blanks (*n* = 3) were ran in parallel to mulch film extraction and analysis to ensure the analytes detected were mulch film derived.

## Results and discussion

The following sections present detailed characterisation of the bulk polyester composition of an agricultural plastic mulch film with ^1^H NMR, and mulch film extract composition with GC, GC-MS, DI-Orbitrap-MS and HPLC-Orbitrap-MS/MS. The methodology is evaluated with the aim of improving the current understanding of the composition of polyester biodegradable mulch films. We show that combining analytical approaches is essential, as each offers unique advantages for revealing different aspects of the organic chemical composition of the film. Lastly, the significance of the presented results with regards to potential environmental implications and wider applications of the developed complementary spectrometric techniques are discussed, highlighting the importance of in-depth chemical interrogation of the composition of biodegradable mulch films.

### Characterisation of the bulk polyester composition with ^1^H NMR

**Fig. 2:**
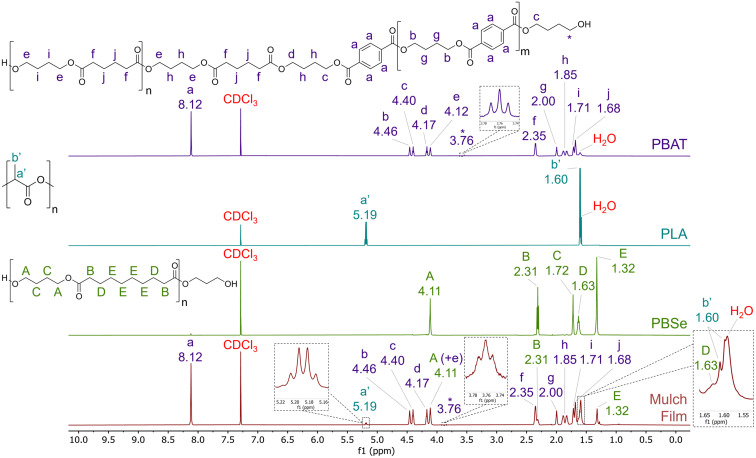
Stacked ^1^H NMR spectra (600 MHz, CDCl_3_, 25 °C) of PBAT, PLA and PBSe standards and PLA/PBAT mulch film. Resonance signals for each polymer standard are assigned to specific ^1^H nuclei environments with a characteristic letter/symbol and correspondingly assigned in the mulch film spectrum. Peaks were assigned for PBAT based on Nifant'ev *et al.*,^49^ de Ilarduya and Muñoz-Guerra^50^ and Nelson *et al.*,^21^ and for PBSe using Siotto *et al.*^51^

To determine the polymeric composition, reported as 85% PBAT and 15% PLA, 1D ^1^H NMR spectra of reference PLA, PBAT and PBSe standards were compared to the PLA/PBAT mulch film (Fig. 2). PBSe was an unreported polyester component of the mulch film, likely as polybutylene sebacate-*co*-terephthalate (PBSeT),^47^ or included as a biodegradable thermoplastic adhesive.^48^ The molar percentages (mol%) and molar weight percentages (mol wt%) of the polymers, along with the assigned ^1^H NMR environment signal intensities and proton numbers used in the calculations, are presented in ESI (Section S6; Table S2).† Due to the possibility of PBSeT being present in the material, PBT contribution to PBAT could not be determined with confidence. The components of PBAT were, therefore, broken down to PBA and PBT and evaluated individually. As expected, PBA and PBT were the dominant polymer constituents in the material at molar percentage of the polymer content of 35 mol% and 40 mol% respectively. When combined with the molar percentage of PBSe (12 mol%), this totals 87 mol%, which aligns with the reported contribution of PBAT (85%); albeit with the absence of PBSe. The molar percentage of PLA was calculated as 13 mol%, however, its molar weight percentage was only 5 mol wt%. This indicates that PLA contributes less to the overall mass of the material compared to the other polyester components: PBA (35 mol wt%), PBT (45 mol wt%) and PBSe (15 mol wt%).

For polyesters, confirming polymeric composition is a key step that informs characterisation of the extractable oligoester content or *vice versa*. Py-GC-MS is another alternative approach, which was successful in this study at characterising the precipitated polymer formed during extraction (Section S2; ESI†) and the pyrolysis products for PLA and PBAT are well documented.^45,52–56^ Crucially, here ^1^H NMR enabled quantitative characterisation of the contribution from an undeclared polyester component (PBSe) which made a comparable contribution to the bulk composition to PLA.

### Organic chemical characterisation of PLA/PBAT mulch film using a range of spectrometric instrumentation and approaches

**Fig. 3:**
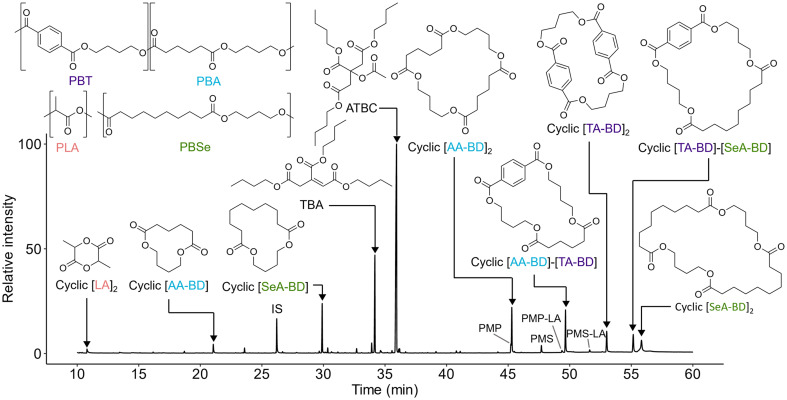
GC-MS TIC (*m*/*z* 15–650) of the dissolution precipitation MeOH extract of the the PLA/PBAT mulch, after MSTFA derivatisation. IS indicated benzyl benzoate. Presented chemical structures include acetyltributyl citrate (ATBC) and its derivative tributyl aconitate (TBA) and cyclic oligoesters. Cyclic oligoesters are abbreviated to their monomeric components: adipic acid (AA), terephthalic acid (TA), sebacic acid (SeA), lactic acid (LA) and 1,4-butanediol (BD). Pentaerythritol monopalmitate (PMP) and pentaerythritol monostearate (PMS) are labelled along with their lactic acid functionalised derivatives PMP-LA and PMS-LA.

Compounds identified with GC-MS are presented in Fig. 3, with further detail on characterisation and quantification presented in the ESI (Section S7; Table S3).† The plasticisers acetyl tributyl citrate (ATBC; 4210 ± 135 μg g^−1^), tributyl citrate (TBC; 104 ± 23 μg g^−1^) and fatty acid lubricants, palmitic acid (18 ± 3 μg g^−1^) and stearic acid (4 ± 2 μg g^−1^), were the only identified additives in extracts. Accompanying ATBC and TBC was the additive derived NIAS tributyl aconitate (TBA; 1400 ± 170 μg g^−1^). Pentaerythritol monopalmitate (PMP) and pentaerythritol monostearate (PMS) and their lactic acid functionalised derivatives were uniquely detected in MSTFA derivatised extracts, and their structural elucidation with GC-QTOF-MS is detailed in the ESI (Section S4).†^57,58^ Possible sources for these components are uncertain without initial compositional knowledge but include derivatives of pentaerythritol fatty ester slip agents/lubricants,^13^ PLA branching agents^59^ or pentaerythritol phosphite PLA hydrolysis aids.^60,61^

Other identified chemical suspects include cyclic polyester derived compounds. Oligoesters are assigned identifiers based on their monomeric constituents. This may be broken down to the following nomenclature: AA – adipic acid, TA – terephthalic acid, SeA – sebacic acid, LA – lactic acid and BD – 1,4-butanediol. The cyclic oligoesters, aside from lactide (cyclic [LA]_2_) and 1,6-dioxacyclododecane-7,12-dione (cyclic [AA-BD]), could not be found through library comparison. Details on their identification are discussed in Monkley *et al.*^37^ Extract content of cyclic oligoesters ranged between 168 ± 32 μg g^−1^ for cyclic [AA-BD] up to 772 ± 5 μg g^−1^ for cyclic [AA-BD]_2_ (Table 1). The combined cyclic oligoester concentration reported here (2900 ± 16 μg g^−1^) is comparable to the total additive content (4370 ± 130 μg g^−1^) stressing the importance of characterising chemical constituents in biodegradable mulches beyond additives alone.

**Fig. 4:**
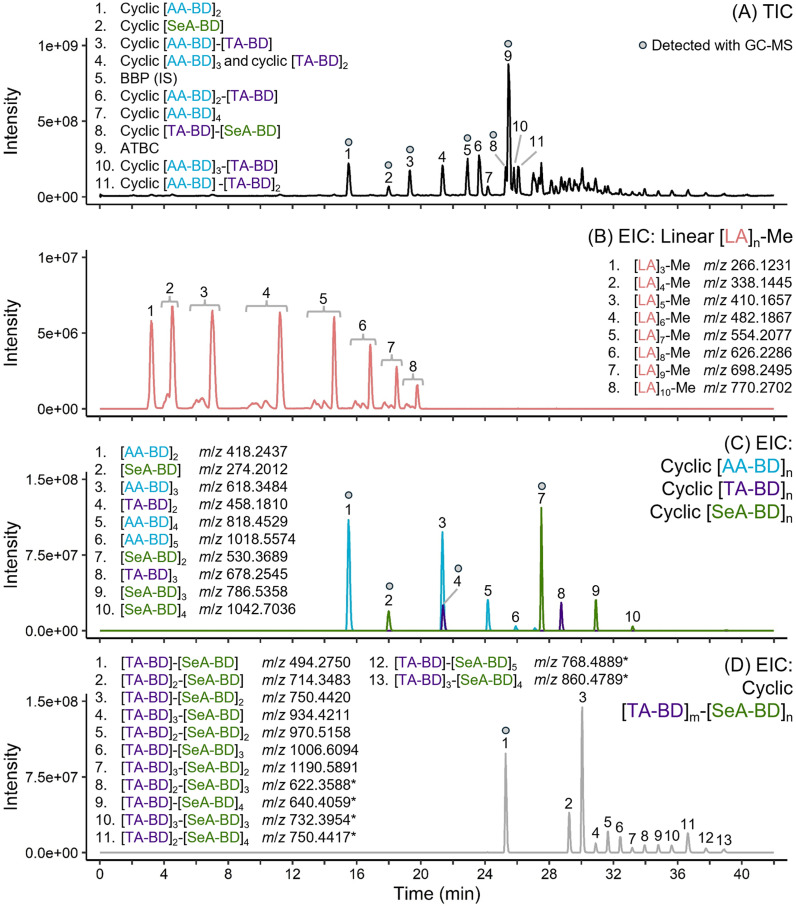
Stacked HPLC-Orbitrap-MS chromatograms of a PLA/PBAT mulch film dissolution-precipitation extract. Presented chromatograms include TIC (*m*/*z* 150–2000) (A) and extracted ion chromatograms (EIC) for oligomer sequences [LA]_*n*_-Me (B), [AA-BD]_*n*_, [TA-BD]_*n*_ and [SeA-BD]_*n*_ (C), and [TA-BD]_*m*_-[SeA-BD]_*n*_ (D). EIC (B), (C) and (D) are presented with numbered peaks where the number corresponds to assigned oligoester structures and the extracted ion *m*/*z*. Number assignment is specific to each individual chromatogram. In all cases, precursor ions are [M + NH_4_]^+^ adducts, except higher molecular weight cyclic [TA-BD]_*m*_-[SeA-BD]_*n*_ where the most abundant precursor ion was [M + 2NH_4_]^2+^ denoted by a *. Circles indicate compounds also detected with GC-MS.

Although no additional unique additives were detected beyond those identified by GC-MS, both DI-Orbitrap-MS and HPLC-Orbitrap-MS/MS revealed additional complexity of the oligomeric components in the PLA/PBAT mulch (Fig. 4). The structural assignment of oligomeric series is presented in Table 1, with detailed overviews for assigned structures in the ESI (Spreadsheet 2–7 and Spreadsheet 9, respectively).† Common precursor adducts for oligoesters were primarily [M + NH_4_]^+^ and for additive components [M + H]^+^. For larger cyclic oligoester components, usually above 6 or more monomer components, [M + 2NH_4_]^2+^ was the most intense precursor ion due to more sites for adduct formation. Along with homopolymeric cyclic oligoester components ([AA-BD]_*n*_ (*n* = 1–6), [TA-BD]_*n*_ (*n* = 2–3), [SeA-BD]_*n*_ (*n* = 1–4) and [LA]_*n*_ (*n* = 6–12)), other monomer combinations were limited to cyclic [AA-BD]_*m*_-[TA-BD]_*n*_ and cyclic [TA-BD]_*m*_-[SeA-BD]_*n*_. The presence of the [TA-BD]_*m*_-[SeA-BD]_*n*_ oligoester sequence (Fig. 4) supports the inclusion of PBSeT in the material. PBAT-derived cyclic oligoesters comprising up to 9 monomeric components were assigned, such as cyclic [AA-BD]_5_-[TA-BD]_4_ and cyclic [AA-BD]_6_-[TA-BD]_3_. The highest molecular weight oligoester component detected with HPLC-Orbitrap-MS was [TA-BD]_3_-[SeA-BD]_4_ as the [M + 2NH_4_]^+^ adduct. Additionally, methyl capped linear PLA oligomers were detected from [LA]_2_-Me up to [LA]_20_-Me through DI-Orbitrap-MS. There was a general trend for all oligoester series (*e.g.* cyclic [AA-BD]_*n*_, cyclic [TA-BD]_*n*_, cyclic [PBSe-BD]_*n*_ and linear [LA]_*n*_-Me) that peak intensities would decrease as size increased (Fig. 4).

Linear [LA]_*n*_-Me components displayed multiple retention times for the same precursor ion with HPLC-Orbitrap-MS, with maximum difference of 1.76 min (Fig. 4). These PLA oligomer components include multiple stereogenic centers along the polymer chain, which means different combinations of *R* and *S* configurations are possible at these sites.^62^ Similarly, some detected oligomer components comprising AA, TA and BD were found to have two or three similar retention times, with maximum difference of 0.42 min. For example, extraction of the precursor ion *m*/*z* 858.3907 for [M + NH_4_]^+^ of cyclic [AA-BD]_2_-[TA-BD]_2_ results in two peaks with retention times of 27.10 min and 27.35 min. This can be attributed to the presence of structural or diastereoisomers and has been observed elsewhere.^62^ Both the specific structure or stereochemistry of each isomer and the eluting peak in the chromatogram they relate to remains unclear.

Biodegradable plastic mulch films represent a potential anthropogenic chemical input source to agricultural soils used for food production. Because of this, material compositional analysis is necessary to inform potential implications for the environment. Complications arise due to the chemical composition of plastic mulch films often being proprietary, requiring investigation through appropriate analytical interrogation to reveal the chemical components of the material. Analogous analytical approaches to that presented herein for NTS of PLA/PBAT solvent extracts have found diverse additive loads, including bisphenols, phenolic antioxidants, organophosphates, and diisocyanates,^30^ and benzotriazoles, benzophenones and HALS.^27,28^ The reporting of methodologies that account for the oligoester contribution to mulch films is less common. Cui *et al.*^33^ detected cyclic [LA]_2_ and quantified cyclic [AA-BD] (97 μg g^−1^) in the MeOH extracts of PLA/PBAT mulch with GC-MS. Reay *et al.*^16^ attributed 37% of the volatile fraction in biodegradable mulch film extracts to oligoesters, including cyclic [AA-BD]_2_, cyclic [AA-BD]-[TA-BD]_2_, and cyclic [TA-BD]_2_, and additionally highlighted their potential to leach from the material to salt solutions. Furthermore, Serrano-Ruiz *et al.*^63^ reported the detection of PLA/PBAT monomer components in the leachate of biodegradable mulches, and suggested other components detected with ^1^H NMR were oligoesters but could not assign them identities. Differences in the additive composition of biodegradable mulches highlight the need for the expansion of NTS methodologies to provide a diagnostic overview of chemical composition. This is especially true for mulch films in use outside of China, given so far the main body of sampling has occurred in this region.^20^ Therefore, this study provides a valuable characterisation workflow to comprehensively detect and assign both additives and the significant oligoester components of PLA/PBAT mulch films across a full molecular weight range.

### Method evaluation

Dissolution-precipitation using DCM–MeOH was performed to extract additives and NIASs from the polymer matrix. This method was selected for its adaptability across different laboratories without requiring specialised equipment and to facilitate the extraction of oligoester components.^13^ Recovery rates for tested compounds ranged from 55–80% (compared to plastic dissolved in DCM and passively dried before extraction) and 67–86% (compared to pristine PLA/PBAT mulch) (Table S1; ESI†). Notably, recovery from the reformed film showed no significant difference from pristine PLA/PBAT mulch (Student's *t*-test, *p* > 0.05), suggesting that pre-dissolution had no impact on extraction efficiency. Cui *et al.*^33^ achieved 75–98% recovery using a similar spiking experiment with microwave-assisted extraction with MeOH, followed by dispersive liquid-liquid microextraction (DLLME), for a diverse range of additives from PLA/PBAT mulches. This included recovery of cyclic [AA-BD] at 86%. In contrast, the 67% recovery for cyclic [AA-BD] observed here may result from occlusion within the precipitated polymer matrix. It is feasible the lower recovery herein is a result of entrapment within the re-formed solid upon polymer precipitation with the antisolvent (MeOH).^13^ An alternative approach is solvent extraction with no dissolution of the matrix, only swelling of the polymer, however, diffusion limitations – including migrant molecular weight and plastic particle size (surface area) – may also impair recovery. Ultimately, the choice of extraction procedure is dependent on the study aim, which in this case was maximising compositional understanding of the material.

GC and GC-MS were selected for their focus on detecting lower MW components, which are most likely to be released and be bioavailable in the environment. In total, 16 components of the mulch were characterised with GC-MS and 12 were quantified by GC-FID with reference or surrogate material (Table S2; ESI†). Although detection of PBAT and PBSeT oligoester components was limited up to dimers (*e.g.* cyclic [AA-BD]_2_, [AA-BD]-[TA-BD], [SeA-BD]_2_, *etc*.), GC-MS successfully detected all major additive components. Furthermore, there was good correspondence between detection of the oligoester components with the highest peak areas found through HPLC-Orbitrap-MS (Fig. 4). The general decrease seen in intensity along oligoester sequences would suggest that cyclic oligoesters towards the lower end of the repeat unit scale (*e.g.* dimers and trimers) are most abundant in the material and/or have higher extraction efficiency. This trend, also reported for oligoesters from lacquers^64^ or those migrating from food packaging materials,^65,66^ highlights the utility of GC-MS for oligoester characterisation.^16,67,68^ To be more conclusive of the decreasing abundance along oligoester series, quantitative determination is required for oligoester components beyond the GC analytical window. Challenges in confirming ionisation efficiency during HESI, due to limited reference materials, could be addressed using predictive algorithms trained on available surrogate data.^69,70^

DI-Orbitrap-MS extended the detection range of oligomer components, but identification confidence was limited to molecular formula assignment without diagnostic fragment ions. Despite this, across 465 assigned ions, the mass accuracy error (*δ*) (difference between detected accurate mass of a given ion and the calculated exact mass for the assigned elemental formula of that ion) ranged from −0.00124 to 0.00278 Da. A total of 106 ion sequences, after refining for duplicates and isotopologues, revealed 97 oligoester components (Spreadsheet 2–7; ESI†). HPLC-Orbitrap-MS analysis characterised 56 mulch film components, including major additives and oligoesters previously detected by GC-MS (Spreadsheet 9; ESI†). Therefore, DI-Orbitrap-MS broadened the detection range of oligoesters beyond traditional peak-by-peak workflows to an additional 38 oligoester components and predominantly aided in the detection of linear PBAT components (BD-[AA-BD]_*m*_-[TA-BD]_*n*_), cyclic PLA oligoesters ([LA]_*n*_, *n* = 6–12) and linear methyl capped PLA oligoesters ([LA]_*n*_-Me (*n* = 11–20)). The use of both DI-Orbitrap-MS and HPLC-Orbitrap-MS/MS is advised given the latter provides diagnostic structural information for both oligoesters and additive components. An alternative complementary approach for HPLC-Orbitrap-MS/MS to extend oligoester characterisation was developed by Omer *et al.*,^62^ which uses molecular networking to computationally compare HPLC-HRMS/MS spectra for filtered precursor ions to find analytes of spectral and structural similarity. This resulted in the unique assignment of PBAT oligoester series as well as the discovery of a new oligomer series comprising azelaic acid in the tested polyester formulations.

### Environmental context and wider application

Additive composition analysis is crucial for evaluating the potential risks biodegradable mulch film co-contaminants may pose to agroecosystems, which may include the transfer of toxic chemicals to agroecosystems.^14,27,28,31^ Hence, many characterisation studies on plastic mulch films have focussed on hazardous additive components of known concern.^27,29,30^ While this study did not identify any concerning additives, aligning with standardised biodegradable mulch film requirements within the EU,^18^ it provides robust, unbiased NTS methodologies for comprehensive assessment, exemplified by the identification of PBSeT and substantial oligoester derivative components in the material. This may be especially important given a significant proportion of plastic chemicals in commercial articles, including mulch films,^16^ are yet to be assigned structural identities or lack sufficient hazard information, limiting our understanding of their associated risk.^71,72^ Many of the oligoesters fall into this category, and despite likely being readily biodegradable as derivatives of biodegradable PBAT and compostable PLA, environmental implications remain unclear. The environmental relevance of oligoesters has been highlighted by Hu *et al.*,^39^ who identified polyester mulch films as concentrated sources of oligomers and monomers in agroecosystems, with their impacts on soil properties, such as pH, or microbial functioning remaining poorly understood. Similarly, Yoshinaga *et al.*^73^ observed that short-chain oligoesters (up to four repeat units) from polycaprolactone exhibited toxicity to marine algae and mammalian cells, whereas longer oligoester chain lengths and polyester were less toxic. Therefore, holistic methodologies that are unbiased in the screening of chemical components, such as that presented herein, are necessary to assess the environmental safety of the chemical burden introduced to agroecosystems through biodegradable mulching practice.

Furthermore, the presented methods for oligoester detection can extend beyond plastic formulation characterisation to molecular-level monitoring of material degradation. This may be achieved by monitoring compositional shifts in oligoester distributions as polyester degradation proceeds over time or under different degradation stressors (*e.g.* UV light, biotic activity, soil moisture content, *etc.*).^38^ There is further scope for the analytical approaches presented here to be adapted for environmental monitoring in leachate, soil, or biomass matrices, providing essential insights for life-cycle assessments of biodegradable mulch films.

## Conclusions

This study presents a comprehensive workflow to characterise the major organic components of PLA/PBAT mulch film through the application of multiple complementary analytical techniques. From the results obtained, the following conclusions can be drawn:

(i) ^1^H NMR successfully attributed the relative contribution each polyester component (PLA, PBA, PBT and PBSe) made to the material, including an undeclared PBSe component likely incorporated as PBSeT. Therefore, ^1^H NMR with comparison to polyester standards or reference materials is suggested as the preferred spectroscopic approach for bulk polyester compositional analysis.

(ii) PLA/PBAT mulch solvent extracts were investigated with GC-MS, DI-Orbitrap-MS (oligomer sequence extraction) and HPLC-Orbitrap-MS/MS. A total of 107 plastic-derived compounds were characterised. NTS with GC-MS and GC-FID is suggested prior to subsequent DI-Orbitrap-MS or HPLC-Orbitrap-MS as it can inform the content of additive and oligoester components, the latter of which can be expected to be of reduced abundance with increasing chain length. For oligoester quantification beyond the GC-amenable window, the production of suitable reference material or standards is required.

(iii) Only through the combination of the presented analytical workflows can the complexity of the oligoester components in biodegradable mulch films be characterised. It is important that the workflow is applied to other PLA/PBAT mulches to comprehensively map the organic chemical composition of such materials, particularly the oligoester contribution. This is of significant importance for improving our current knowledge on the chemical burden introduced to agroecosystems through biodegradable plastic mulch film practice.

(iv) There is additional scope to apply the combined analytical approach to monitor changes in oligoester distribution during the biodegradation of mulch films or release of components to surrounding agroecosystem compartments. This can ultimately inform life cycle assessment of the material at the chemical level.

## Conflicts of interest

The authors declare that they have no known competing financial interests or personal relationships that could have appeared to influence the work reported in this paper.

## Supplementary Material

AN-150-D5AN00423C-s001

AN-150-D5AN00423C-s002

## Data Availability

The data supporting this article have been included as part of the ESI.†

## References

[cit1] Kader M. A., Senge M., Mojid M. A., Ito K. (2017). Soil Tillage Res..

[cit2] Yu Y.-Y., Turner N. C., Gong Y.-H., Li F.-M., Fang C., Ge L.-J., Ye J.-S. (2018). Eur. J. Agron..

[cit3] Gao H. H., Yan C. R., Liu Q., Ding W. L., Chen B. Q., Li Z. (2019). Sci. Total Environ..

[cit4] Kader M. A., Singha A., Begum M. A., Jewel A., Khan F. H., Khan N. I. (2019). Bull. Natl. Res. Cent..

[cit5] Wang K., Liu X., Chadwick D. R., Yan C., Reay M., Ge T., Ding F., Wang J., Qi R., Xiao M., Jiang R., Chen Y., Ma J., Lloyd C., Evershed R. P., Luo Y., Zhu Y., Zhang F., Jones D. L. (2025). Environ. Int..

[cit6] Zhang D., Mak-Mensah E., Zhou X., Wang Q., Obour P. B. (2023). J. Soil Sci. Plant Nutr..

[cit7] Rizzarelli P., Rapisarda M., Ascione L., Degli Innocenti F., La Mantia F. P. (2021). Polym. Degrad. Stab..

[cit8] Li S., Ding F., Flury M., Wang Z., Xu L., Li S., Jones D. L., Wang J. (2022). Environ. Pollut..

[cit9] Yang Y., Li Z., Yan C. R., Chadwick D., Jones D. L., Liu E. K., Liu Q., Bai R. H., He W. Q. (2022). Sci. Total Environ..

[cit10] Serrano-Ruiz H., Martin-Closas L., Pelacho A. M. (2021). Sci. Total Environ..

[cit11] Wang Z., Li M., Flury M., Schaeffer S. M., Chang Y., Tao Z., Jia Z., Li S., Ding F., Wang J. (2021). Sci. Total Environ..

[cit12] Samphire M., Chadwick D. R., Jones D. L. (2023). Front Agron..

[cit13] Zweifel H. , Maier R. D. and Schiller M., Plastic Additives Handbook, Hanser Publishers, Munich, 6th edn., 2009

[cit14] Hahladakis J. N., Velis C. A., Weber R., Iacovidou E., Purnell P. (2018). J. Hazard. Mater..

[cit15] Bridson J. H., Gaugler E. C., Smith D. A., Northcott G. L., Gaw S. (2021). J. Hazard. Mater..

[cit16] Reay M. K., Graf M., Murphy M., Li G., Yan C., Bhattacharya M., Osbahr H., Ma J., Chengtao W., Shi X., Ren S., Cui J., Collins C., Chadwick D., Jones D. L., Evershed R. P., Lloyd C. E. M. (2025). J. Hazard. Mater..

[cit17] Zhou J., Jia R., Brown R. W., Yang Y., Zeng Z., Jones D. L., Zang H. (2023). J. Hazard. Mater..

[cit18] EN 17033 , Plastics - Biodegradable mulch films for use in agriculture and horticulture - Requirements and test methods. European Committee For Standardization, Brussels, Belgium, 2018

[cit19] ECHA

[cit20] Cao X., Liang Y., Jiang J., Mo A., He D. (2023). TrAC, Trends Anal. Chem..

[cit21] Nelson T. F., Remke S. C., Kohler H.-P. E., McNeill K., Sander M. (2020). Environ. Sci. Technol..

[cit22] Sintim H. Y., Bary A. I., Hayes D. G., Wadsworth L. C., Anunciado M. B., English M. E., Bandopadhyay S., Schaeffer S. M., DeBruyn J. M., Miles C. A., Reganold J. P., Flury M. (2020). Sci. Total Environ..

[cit23] Álvarez-Méndez S. J., Ramos-Suárez J. L., Ritter A., Mata González J., Camacho Pérez Á. (2023). Heliyon.

[cit24] Rizzarelli P., Rapisarda M., Perna S., Mirabella E. F., La Carta S., Puglisi C., Valenti G. (2016). J. Anal. Appl. Pyrolysis.

[cit25] Akoueson F., Chbib C., Monchy S., Paul-Pont I., Doyen P., Dehaut A., Duflos G. (2021). Sci. Total Environ..

[cit26] Khatsee S., Daranarong D., Punyodom W., Worajittiphon P. (2018). J. Appl. Polym. Sci..

[cit27] Fan R., Li B., Liu Q., Liu Q., Cui J., Bai R., Wang Y., Elias R., Li C., He W. (2024). J. Hazard. Mater..

[cit28] Li B., Liu Q., Yao Z., Ma Z., Li C. (2023). Environ. Pollut..

[cit29] Gong X. Y., Zhang W. J., Zhang S. Y., Wang Y., Zhang X. Y., Lu Y., Sun H. W., Wang L. (2021). Environ. Sci. Technol..

[cit30] Xu Y., Zeng L., Tao Y., Xu J., He Y., Lu Z. (2023). Environ. Sci. Technol..

[cit31] Li C., Chen J. Y., Wang J. H., Han P., Luan Y. X., Ma X. P., Lu A. X. (2016). Sci. Total Environ..

[cit32] Scopetani C., Selonen S., Cincinelli A., Pellinen J. (2023). Front. Environ. Sci..

[cit33] Cui H., Gao W. C., Lin Y. C., Zhang J., Yin R. S., Xiang Z. M., Zhang S., Zhou S. P., Chen W. S., Cai K. (2021). Microchem. J..

[cit34] Goodman I., Nesbitt B. F. (1960). Polymer.

[cit35] Vermylen V., Lodefier P., Devaux J., Legras R., Mac Donald W. A., Rozenberg R., De Hoffmann E. (2000). J. Polym. Sci., Part A: Polym. Chem..

[cit36] Ubeda S., Aznar M., Nerín C. (2018). Anal. Bioanal. Chem..

[cit37] Monkley C., Reay M. K., Evershed R. P., Lloyd C. E. M. (2024). Rapid Commun. Mass Spectrom..

[cit38] Sander M. (2019). Environ. Sci. Technol..

[cit39] Hu L., Zhou Y., Chen Z., Zhang D., Pan X. (2023). Environ. Sci. Technol..

[cit40] Izunobi J. U., Higginbotham C. L. (2011). J. Chem. Educ..

[cit41] Chambers M. C., Maclean B., Burke R., Amodei D., Ruderman D. L., Neumann S., Gatto L., Fischer B., Pratt B., Egertson J., Hoff K., Kessner D., Tasman N., Shulman N., Frewen B., Baker T. A., Brusniak M.-Y., Paulse C., Creasy D., Flashner L., Kani K., Moulding C., Seymour S. L., Nuwaysir L. M., Lefebvre B., Kuhlmann F., Roark J., Rainer P., Detlev S., Hemenway T., Huhmer A., Langridge J., Connolly B., Chadick T., Holly K., Eckels J., Deutsch E. W., Moritz R. L., Katz J. E., Agus D. B., MacCoss M., Tabb D. L., Mallick P. (2012). Nat. Biotechnol..

[cit42] Pemberton J. A. , PhD Doctoral Thesis, University of Bristol, 2018

[cit43] Gatto L., Gibb S., Rainer J. (2021). J. Proteome Res..

[cit44] Morgan M. and Ramos M.. BiocManager: Access the Bioconductor Project Package Repository, 2024. https://CRAN.R-project.org/package=BiocManager

[cit45] R Core Team , R: A Language and Environment for Statistical Computing, R Foundation for Statistical Computing, Vienna, Austria, 2022. https://www.R-project.org/

[cit46] Schymanski E. L., Jeon J., Gulde R., Fenner K., Ruff M., Singer H. P., Hollender J. (2014). Environ. Sci. Technol..

[cit47] Heidarzadeh N., Rafizadeh M., Taromi F. A., del Valle L. J., Franco L., Puiggalí J. (2017). Polym. Degrad. Stab..

[cit48] Sipol®, Technipol® Bio 707, https://www.sipol.com/en/biodegradable-polymers-technipol-bio/technipol-bio-707/, (accessed 12th December 2022)

[cit49] Nifant'ev I. E., Bagrov V. V., Komarov P. D., Ilyin S. O., Ivchenko P. V. (2022). Polymers.

[cit50] de Ilarduya A. M., Muñoz-Guerra S. (2014). Macromol. Chem. Phys..

[cit51] Siotto M., Zoia L., Tosin M., Degli Innocenti F., Orlandi M., Mezzanotte V. (2013). J. Environ. Manage..

[cit52] De Falco F., Nacci T., Durndell L., Thompson R. C., Degano I., Modugno F. (2023). J. Anal. Appl. Pyrolysis.

[cit53] Coralli I., Rombolà A. G., Fabbri D. (2024). J. Anal. Appl. Pyrolysis.

[cit54] Khabbaz F., Karlsson S., Albertsson A.-C. (2000). J. Appl. Polym. Sci..

[cit55] Westphal C., Perrot C., Karlsson S. (2001). Polym. Degrad. Stab..

[cit56] Dhahak A., Grimmer C., Neumann A., Rüger C., Sklorz M., Streibel T., Zimmermann R., Mauviel G., Burkle-Vitzthum V. (2020). Waste Manage..

[cit57] Curstedt T. (1974). Biochim. Biophys. Acta, Lipids Lipid Metab..

[cit58] Harvey D. J., Vouros P. (2020). Mass Spectrom. Rev..

[cit59] Hastenreiter L. L. G., Ramamoorthy S. K., Srivastava R. K., Yadav A., Zamani A., Åkesson D. (2020). Polymers.

[cit60] Ortuoste N., Allen N. S., Papanastasiou M., McMahon A., Edge M., Johnson B., Keck-Antoine K. (2006). Polym. Degrad. Stab..

[cit61] Polidar M., Metzsch-Zilligen E., Pfaendner R. (2022). Polymers.

[cit62] Omer E., Bakiri A., Hammel Y.-A., Sanders M. J., Koster S., Ciclet O. (2025). Food Chem..

[cit63] Serrano-Ruiz H., Eras J., Martin-Closas L., Pelacho A. M. (2020). Polym. Degrad. Stab..

[cit64] Omer E., Cariou R., Remaud G., Guitton Y., Germon H., Hill P., Dervilly-Pinel G., Le Bizec B. (2018). Anal. Bioanal. Chem..

[cit65] Ubeda S., Aznar M., Alfaro P., Nerín C. (2019). Anal. Bioanal. Chem..

[cit66] Osorio J., Aznar M., Nerín C., Elliott C., Chevallier O. (2022). Anal. Bioanal. Chem..

[cit67] Omer E., Bichon E., Hutinet S., Royer A.-L., Monteau F., Germon H., Hill P., Remaud G., Dervilly-Pinel G., Cariou R., Le Bizec B. (2019). J. Chromatogr., A.

[cit68] Ubeda S., Aznar M., Nerín C. (2019). Food Chem..

[cit69] Hollender J., Schymanski E. L., Ahrens L., Alygizakis N., Béen F., Bijlsma L., Brunner A. M., Celma A., Fildier A., Fu Q., Gago-Ferrero P., Gil-Solsona R., Haglund P., Hansen M., Kaserzon S., Kruve A., Lamoree M., Margoum C., Meijer J., Merel S., Rauert C., Rostkowski P., Samanipour S., Schulze B., Schulze T., Singh R. R., Slobodnik J., Steininger-Mairinger T., Thomaidis N. S., Togola A., Vorkamp K., Vulliet E., Zhu L., Krauss M. (2023). Environ. Sci. Eur..

[cit70] Malm L., Liigand J., Aalizadeh R., Alygizakis N., Ng K., Frøkjær E. E., Nanusha M. Y., Hansen M., Plassmann M., Bieber S., Letzel T., Balest L., Abis P. P., Mazzetti M., Kasprzyk-Hordern B., Ceolotto N., Kumari S., Hann S., Kochmann S., Steininger-Mairinger T., Soulier C., Mascolo G., Murgolo S., Garcia-Vara M., López de Alda M., Hollender J., Arturi K., Coppola G., Peruzzo M., Joerss H., van der Neut-Marchand C., Pieke E. N., Gago-Ferrero P., Gil-Solsona R., Licul-Kucera V., Roscioli C., Valsecchi S., Luckute A., Christensen J. H., Tisler S., Vughs D., Meekel N., Talavera Andújar B., Aurich D., Schymanski E. L., Frigerio G., Macherius A., Kunkel U., Bader T., Rostkowski P., Gundersen H., Valdecanas B., Davis W. C., Schulze B., Kaserzon S., Pijnappels M., Esperanza M., Fildier A., Vulliet E., Wiest L., Covaci A., Macan Schönleben A., Belova L., Celma A., Bijlsma L., Caupos E., Mebold E., Le Roux J., Troia E., de Rijke E., Helmus R., Leroy G., Haelewyck N., Chrastina D., Verwoert M., Thomaidis N. S., Kruve A. (2024). Anal. Chem..

[cit71] Wiesinger H., Wang Z., Hellweg S. (2021). Environ. Sci. Technol..

[cit72] Wagner M. , Monclús L., Arp H. P. H., Groh K., J., Løseth M. E., Muncke J., Wang Z., Wolf R. and Zimmermann L., State of the science on plastic chemicals - Identifying and addressing chemicals and polymers of concern, 2024. 10.5281/zenodo.10701706

[cit73] Yoshinaga N., Tateishi A., Kobayashi Y., Kubo T., Miyakawa H., Satoh K., Numata K. (2023). Biomacromolecules.

