# Supplementary material for: Characterisation of the unknown chemical composition of a commercial biodegradable agricultural plastic mulch film using complementary spectrometric and spectroscopic techniques
Source: Analyst. 2025 Jul 3;150(15):3319–32. doi: 10.1039/d5an00423c (PMC12235505; doi:10.1039/d5an00423c)
Supplement: AN-150-D5AN00423C-s002 [file AN-150-D5AN00423C-s002.pdf]

Supplementary Information (SI) for Analyst.
This journal is © The Royal Society of Chemistry 2025

Characterisation of the unknown chemical composition of a commercial biodegradable agricultural plastic mulch film using complimentary spectrometric and spectroscopic techniques

Supplementary Information

Charlie Monkley[a,], Michaela K. Reay[a], Helen Whelton[a], Richard P. Evershed[a], Charlotte E.M. Lloyd[a,b,*]*

[a]*Organic Geochemistry Unit, School of Chemistry, University of Bristol, Cantock's Close, Bristol, BS8 1TS, UK.*

[b]*School of Geographical Sciences, University of Bristol, University Road, Bristol, BS8 1SS, UK.*

[*]Authors for correspondence: Charlotte E.M. Lloyd email: charlotte.lloyd@bristol.ac.uk Tel: +44 (0)117 455 0991, Charlie Monkley email: cm1776@bristol.ac.uk

S.1 *Chemicals and reagents*

Solvents (methanol (MeOH), dichloromethane (DCM), ethyl acetate, acetone, *n*-hexane) were HPLC grade at a purity of >95% (Rathburn Chemicals Ltd). Solvents used as mobile phases for analytical instrumentation were UHPLC-MS grade (≥99.9% purity) and purchased from Thermo Fisher Scientific™ and included MeOH, H_2O and acetonitrile (ACN). Optima™ LC/MS grade ammonium acetate and formic acid were purchased from Fisher Scientific.

The following laboratory reference compounds were purchased from Merck Life Science UK Limited: acetyltributyl citrate (ATBC) (CAS 77-90-7, >98%), 3,6-dimethyl-1,4-dioxane-2,5-dione (CAS 95-96-5, 99%), palmitic acid (CAS 57-10-3, >99%), stearic acid (57-11-4, >99%) and pentaerythritol monostearate (PMS) (CAS 78-23-9, >98%). 1,6-Dioxacyclododecane-7,12-dione (CAS 777-95-7, 98%), 1,6,13,18-tetraoxacyclotetracosane-7,12,19,24-tetraone (CAS 78837-87-3), pharacine (CAS 63440-93-7, 98%) and cyclotris(1,4-butylene terephthalate) (CAS 63440-94-8, >98%) were purchased from Insight Biotechnology.

A standard of PLA (Mw ~60,000 Da; 3 mm granule size) was purchased from Sigma-Aldrich, Merck Life Sciences UK Ltd. A pelletised standard of PBAT was provided by the BioComposites Centre at Bangor University. Sipol® S.p.A. Società Italiana Polimeri provided a sample of their adhesive product Technipol® Bio 707 to use as a reference standard for polybutylene sebacate (PBSe).[1]

S.2 *Characterising unknown "white precipitate" with Py-GC-MS*

S2.1 *Py-GC-MS Instrument Conditions*

PLA standard (58 μg), PBAT standard (144 μg) and the dried unknown "white precipitate" (151 μg) from PLA/PBAT mulch solvent extracts were weighed into pyrolysis tubes and held in place with quartz wool.

The pyrolysis-GC-MS (Py-GC-MS) system comprised of a CDS Analytical LLC Pyroprobe 6200 model, connected to a Thermo Fisher Scientific™ Trace™ 1310 Gas Chromatograph and Thermo Fisher Scientific™ ISQ™ 7000 Single Quadrupole Mass Spectrometer. The pyrolysis tubes were manually placed in the pyroprobe chamber which linked to the GC-MS via a helium (He) transfer line. The pyroprobe instrument settings maintained a constant interface temperature of 300 °C, operating under trap mode. The pyroprobe temperature ramped from 0 °C at a rate of 20 °C ms^{-1} up to 500 °C, which was held for 25 s. The trap temperature at rest was maintained at 50 °C and reached a final desorption temperature of 300 °C (4 min) and the transfer line temperate to the GC was maintained at 310 °C. The GC was fitted with a non-polar Agilent HP-1 column (60 m × 0.32 mm, 0.25 μm film thickness). The column temperature was maintained at 40 °C (6 min), before ramping at 20 °C min^{-1} up to 310 °C (15 min). The GC operated under split injection mode at a split ratio of 20:1 and the He carrier gas maintained a constant flow rate (2 mL min^{-1}). The MS operated under EI (70 eV) at a full scan range (m/z 50 – 650) and the MS transfer line and ion source temperature were maintained at 310 °C.

Pyrolysis tubes were furnaced (1000 °C, 4 h) and contact with the tubes was only made with solvent sterilised metal equipment. Blanks were performed between sample runs at elevated pyroprobe temperatures of 610 °C to ensure no carry over between samples.

S2.2 *Characterisation of the unknown "white precipitate" through Py-GC/MS*

Following solvent extraction and filtration, "white precipitate" remained suspended in the solvent, and this became more apparent as the solution was concentrated. To characterise this, 150 μg of the dry "white precipitate" was pyrolysed at 500 °C and analysed by Py-GC-MS for comparison against PLA and PBAT standard.

Based on the findings of Khabbaz*, et al.* [2] and Westphal*, et al.* [3], peak assignment of the major pyrolysates for PLA was achieved (Figure S1). Lactide, cyclic oligomers and 2,3-pentanedione identification in the pyrogram of the "white precipitate" confirms PLA as a component. Some PLA indicator compounds that form at low concentrations under thermal degradation could not be detected in the pyrogram of the "white precipitate" as PLA makes up only 5 wt. % of the polymer content in the PLA/PBAT blend (*see* Section S7). Many peaks in Figure S1 could not be assigned an identity. Peaks 2, 6 and 9 are related compounds with increasing Mw based on the similarity in the

mass spectra / characteristic ions, perhaps linear dimers, trimers *etc.* Peaks 5 and 8 are also similar but follow a linear hydrocarbon fragmentation pattern suggesting an unsaturated C chain within the compounds structure. This is an unexpected product of PLA and may, therefore, result from additive or a contaminant present in the standard.

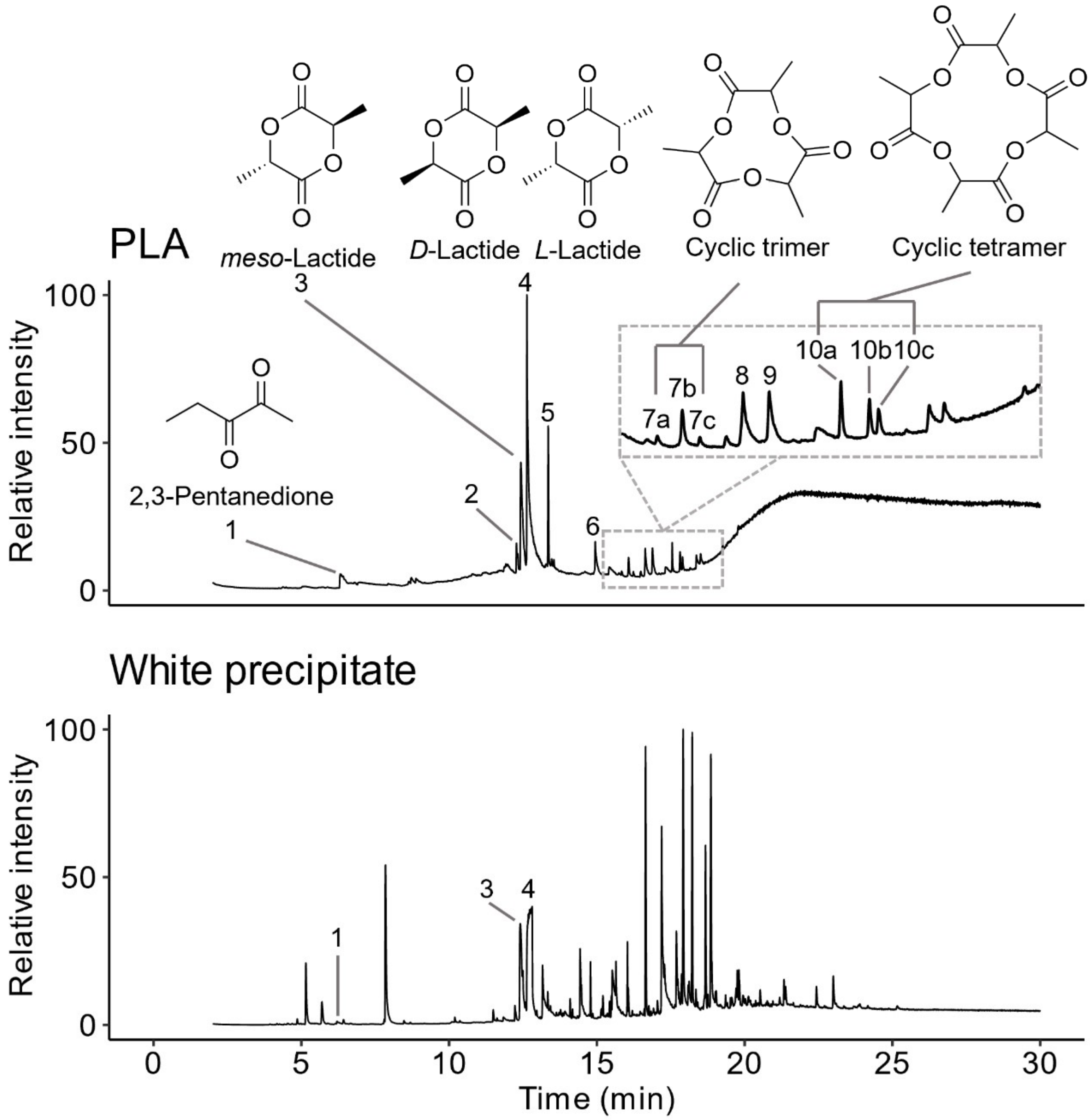

Label	Identity	Characteristic ions / *m/z*
1	2,3-Pentanedione	**57**, 100
2	*Unknown*	**55**, 99, 100, 128
3	meso-Lactide	**56**, 145
4	*D*-Lactide<br>*L*-Lactide	**56**, 145
5	Unknown	**55**, 69, 83, 97, 111, 125, 140, 168
6	Unknown	**55**, 57, 99, 127, 145
7	Unknown	**55**, 99, 100, 128
8	Unknown	**55**, 69, 73, 83, 97, 125, 140, 168
9	Unknown	**55**, 99, 127, 174, 199

Figure S1: Pyrogram (*m/z* 15 – 650) of PLA standard (Top) and the unidentified "white precipitate" from MeOH extracts (Bottom). Labelled peaks are presented with characteristic ions where bold represents the base peak. Peak assignment was adapted from the work of Khabbaz*, et al.* [2] and Westphal*, et al.* [3].

Figure S2 compares the pyrograms of PBAT standard (Top) and the "white precipitate" (Bottom). Accordance in the pyrolytic products between the two spectra confirms that PBAT is a major component of the "white precipitate". Major pyrolysis products are sourced from either PBA or PBT segments of the co-polymer or a combination of the two. The characteristic terephthalic acid fragment ion $[C_8H_5O_3]^+$ (*m/z* 149) was chosen to indicate pyrolysates sourced from PBT,[4,5] and the adipic acid fragment ion $[C_6H_9O_3]^+$ (*m/z* 129) determined the pyrolysis products origin as PBA derived. Some eluting compounds could be tentatively characterised with reference to library spectra with a structural match of ≥90% including: tetrahydrofuran (THF) (NIST Match Factor: 94%), benzene (93%), cyclopentanone (93%), 3-butenyl pentanoate (90%), benzoic acid (92%), 1,6-dioxacyclododecane-7,12-dione (cyclic [AA-BD]; 92%) and di(3-butenyl) terephthalate (90%). Peak 6, 7 and 9 are suspected as 3-butenyl benzoate, 3-butenyl adipate and di(3-butenyl) adipate respectively, due to correspondence in retention times and characteristic ions presented by De Falco*, et al.*[5]. Similarly, the fragment ions of peak 20 match those cyclic [AA-BD]-[TA-BD], previously identified in solvent extracts.[6] Full characterisation of the remaining pyrolysates was beyond the focus of this study but is explored in more depth by Coralli*, et al.*[7].

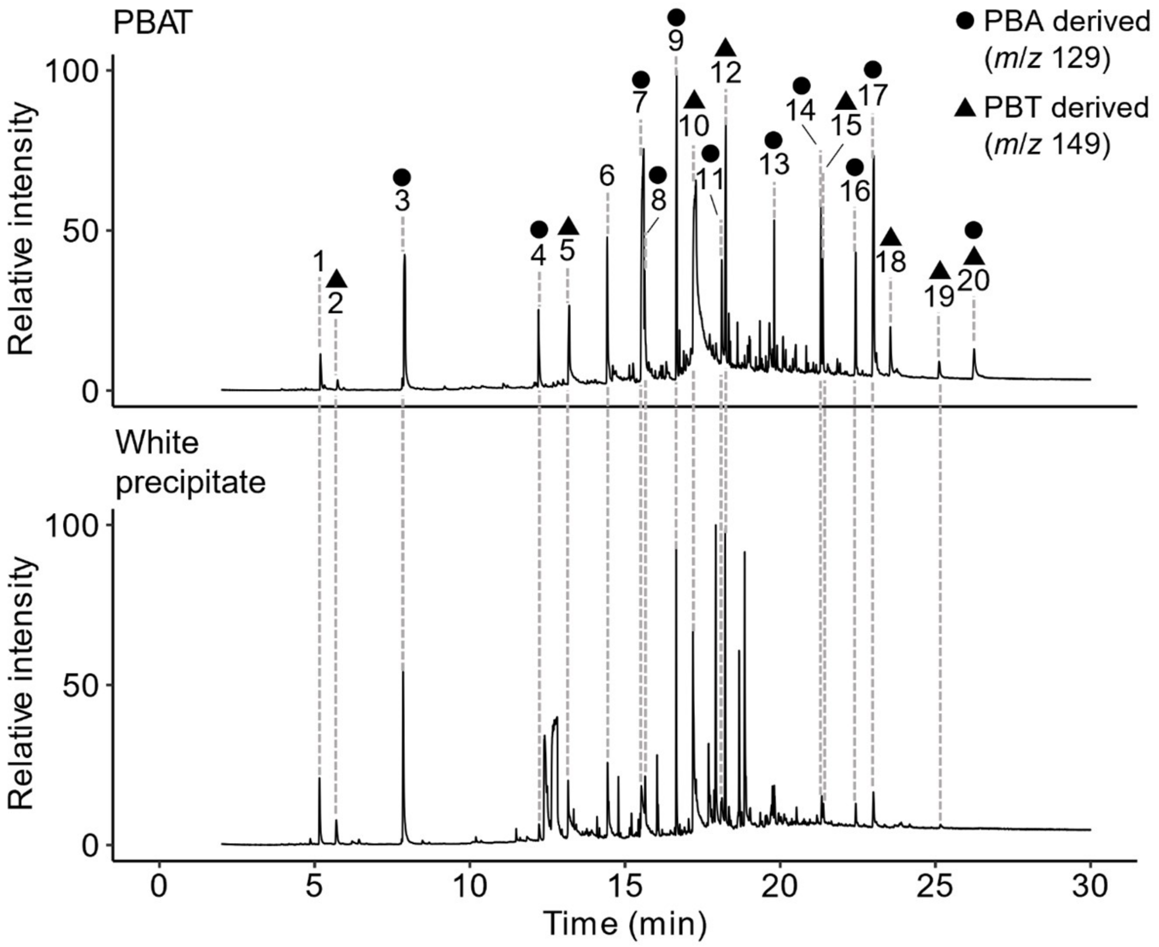
PBAT
White precipitate
Relative intensity
Time (min)
PBA derived (m/z 129)
PBT derived (m/z 149)

94

Label	Identity	NIST MF / %	Characteristic ions / *m/z*
1	Tetrahydrofuran (THF)	97	71, **72**
2	Benzene	91	77, **78**
3	Cyclopentanone	93	**55**, 84
4	3-Butenyl pentanoate	94	54, 57, **85**, 115
5	Benzoic acid	94	51, 77, **105**, 122
6	*3-Butenyl benzoate*	-	54, 77, **105**, 122, 176
7	*3-Butenyl adipate*	-	**54**, 55, 83, 101, 111, 129
8	Cyclic PBA monomer	92	**55**, 71, 73, 84, 100, 111, 129
9	*Di(3-butenyl) adipate*	-	**55**, 83, 101, 111, 129, 183
10	*Unknown*	-	54, 65, 76, 104, 120, **149**, 166
11	*Unknown*	-	**55**, 101, 111, 129, 183, 201
12	Di(3-butenyl) terephthalate	90	54, 65, 104, 121, 149, **203**
13	*Unknown*	-	**55**, 111, 129, 157, 183, 201, 243, 285
14	*Unknown*	-	54, 77, **105**, 111, 123, 129, 177, 183
15	*Unknown*	-	**54**, 85, 104, 121, 149, 203, 305
16	*Unknown*	-	**55**, 84, 101, 111, 129, 141, 183, 201, 227
17	*Unknown*	-	**55**, 101, 111, 129, 183, 201, 311, 383
18	*Unknown*	-	54, **105**, 121, 149, 176, 203, 220, 325
19	*Cyclic [AA-BD]-[TA-BD]*	-	55, 104, 111, 132, **149**, 221, 320, 420
20	*Unknown*	-	104, 111, 129, 149, 183, **203**, 221, 331

Figure S2: Pyrogram (*m/z* 15 – 650) of PBAT standard (Top) and the unidentified "white precipitate" from MeOH extracts (Bottom). Labelled peaks are presented with characteristic ions where bold represents the base peak. Assignment as PBA or PBT derived is possible based on the characteristic fragment ions of each polymer.

Some major peaks in the pyrogram of the "white precipitate" were not characteristic of PLA or PBAT (Figure S3). Palmitic acid and stearic acid are suspected as unrecovered additives remaining in the polymer residue. Peaks a, b, c and e are tentatively identified as alkenes, speculated as pyrolysis products of unrecovered additives with long hydrocarbon chain character such as fatty acids. Tridecane is also tentatively identified which may be a pyrolysis product of a larger additive or a lubricant.[8] Peak f returns high spectral matches (>90%) with numerous fatty alcohols and alkenes but it was uncertain which specific compound the spectrum could be attributed to. The high structural matches came due to consistencies in hydrocarbon chain fragmentation indicative of apolar compounds. Tentatively identified compounds are also predominantly apolar and this may explain their retention to the polymer residue given extraction was performed with polar MeOH. Peak g (dibut-3-enyl sebacate) can be considered as PBSe derived based on its correspondence in characteristic ions; most notably for *m/z* 185 which is a common fragment of the sebacic acid moiety.

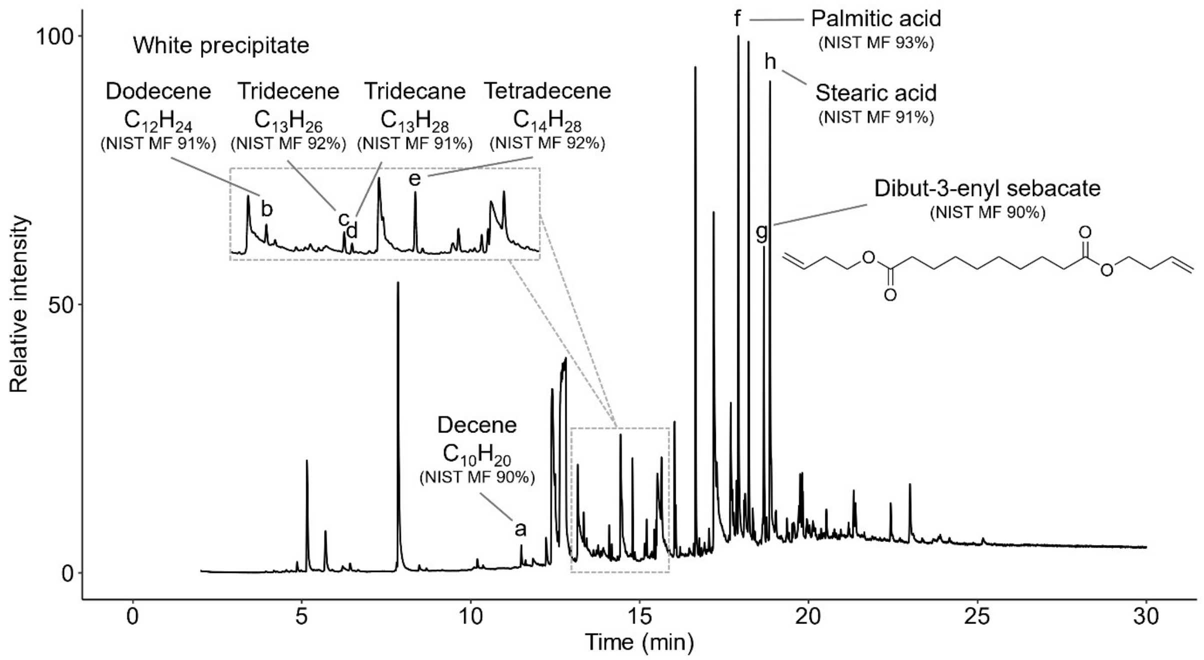

Figure S3: Pyrogram (m/z 15 – 650) of the unidentified "white precipitate" from MeOH extracts. Where possible, peaks are tentatively identified based on spectral library match factors above 90%.

S3. *Analyte recovery assessment*

Recovery assessment was performed through a spiking experiment where PLA/PBAT mulch (0.1 g, n = 3) was dissolved in 3 mL DCM in a glass petri dish. Known amounts of additives and NIASs identified as constituents of the mulch film were added. The added amount (15 – 420 µg) corresponded to the individual content quantified in untreated film extracts (Table S2). The petri dishes were covered in aluminium foil and dried at room temperature. The reformed film was extracted by the dissolution-precipitation extraction procedure. To assess the dissolution treatment on the film, that changes the mulches morphology, triplicate reformed plastic mulches (*i.e.* 0.1 g film dissolved in DCM allowed to reform following DCM evaporation) were also extracted without the addition of spiked reference compounds.

Relative recovery for individual analytes was calculated by:

$$Relative\ recovery\ \% = \frac{C_{spiked\ mulch} - C_{mulch}}{C_{spike}}$$

Where $C_{spiked\ mulch}$ is the extracted content of a given compound from the spiked mulch film, C_{mulch} is the extracted content from untreated mulch and C_{spike} is the added content spiked into dissolved film. C_{mulch} in the above equation was also replaced for $C_{reformed\ mulch}$, where $C_{reformed\ mulch}$ represents the concentration of a given compound in the reformed film that is treated by the same dissolution process as the spiked mulch. Statistical tests for variance (Bartlett), normality (Shapiro-Wilk) and

significant difference between means (Student's t test for two independent data sets) were all computed in R (RStudio 2023.12.0).

Table S1: Relative recovery from standard spiked PLA/PBAT mulch film.

Compound	$Added_{spiked\ film}$ / µg	$Extracted_{spiked\ film}$ / µg	$Extracted_{reformed\ mulch}$ / µg	$RR_{reformed\ mulch}$ / %	$Extracted_{PLA/PBAT\ mulch}$ / µg	$RR_{PLA/PBAT\ mulch}$ / %
Cyclic [AA-BD]	15.4	27.3 ± 1.84	18.8 ± 3.46	55	16.9 ± 3.22	67
ATBC	420	787 ± 13.1	449 ± 54.9	80	424 ± 11.1	86
Cyclic $[AA\text{-}BD]_2$	80.4	134 ± 3.44	71.9 ± 8.61	77	77.7 ± 0.90	70
Cyclic $[TA\text{-}BD]_2$	40.6	62.6 ± 1.13	32.4 ± 4.02	74	35.0 ± 0.83	68
Benzyl benzoate (IS)	60.4	45.5 ± 5.40	39.2 ± 4.41	-	40.5 ± 0.79	-

$RR_{reformed\ mulch}$ – average relative recovery from the spiked film in reference to the reformed mulch film (dissolved in DCM and dried prior to extraction).

$RR_{PLA/PBAT\ mulch}$ – average relative recovery from the spiked film in reference to the PLA/PBAT mulch.

Concentrations are expressed as the mean (n = 3) ± 1 standard deviation.

S4. *Unknown characterisation with GC-QTOF-MS*

S4.1 *GC-QTOF-MS instrument conditions*

GC-QTOF-MS was required for the characterisation of PLA/PBAT mulch film components that could not be identified through MS library comparison. A single representative extract was analysed. Pentaerythritol monostearate (PMS) was additionally derivatised with MSTFA and prepared at 0.01 mg mL^{-1} in ethyl acetate for compound confirmation.

The GC-HRMS system comprised an Agilent Technologies 7890B GC in tandem with an Agilent Technologies 7200 Accurate-Mass Quadrupole Time-of-Flight (Q-TOF) GC/MS. The GC Agilent Technologies 7693 Autosampler injected 1 µL of sample into the multimode inlet (MMI), set to spitless mode with heater temperature setpoint at 70 °C, ramping to 300 °C at 900 °C min^{-1}. The GC column was an Agilent HP-1 (50 m × 0.32 mm, 0.17 µm film thickness) with He carrier gas set to a constant flow rate (1.5 mL min^{-1}). Oven temperature control had starting temperature of 50 °C (2 min), which ramped to 300 °C at 5 °C min^{-1} and was held at this temperature for 15 min. Ion source settings were set to EI at 230 °C with MS acquisition in profile mode ranging from *m/z* 50 – 1050 at scan rate of 5 spectra s^{-1}. Extracts were analysed at low (20 eV) and high (70 eV) collision energies. Data analysis was performed in Agilent's MassHuner Workstation Software (version B.07.00) and elemental composition assignment was achieved using the in-built formula calculator tool. Additional online MS libraries were consulted for unknown identification, including MassBank and ChemSpider, through direct input of HRMS data.

S4.2 *Unknown (Ua – Ud) characterisation with GC-QTOF-MS*

Four compounds detected with GC-MS (Ua – Ud) could not be assigned identities through NIST library comparison and were distinct from oligomer components. Possible structural moieties could initially be inferred due to Ua and Ub returning a 66 – 68% NIST library match with pentaerythritol, 4 trimethylsilyl (TMS) derivative. The mass spectra of Uc and Ud were closely related to Ua and Ub but with a distinct base peak at *m/z* 117, indicative of TMS functionalised carboxylic acid group or secondary alcohol.[9] HRMS data was required for further structural assignment.

Initial focus was placed on the structural derivation of Ua and Ub (Figure S4). The accuracy of fragment ion assignment in the following discussion is represented by δ in Da, which expresses the difference between the detected accurate mass of a given ion and the calculated exact mass for the assigned elemental formula of that ion. Common fragment ions with pentaerythritol, tetrakis-TMS derivative, such as *m/z* 191.0919 ($[C_7H_{19}O_2Si_2]^+$; δ = −0.0001 Da), *m/z* 147.0641 ($[C_5H_{15}OSi_2]^+$; δ = −0.0015 Da) and *m/z* 73.0455 ($[C_3H_9Si]^+$; δ = −0.0013 Da), were assigned with reference to the work of Harvey and

Vouros [9] and Curstedt [10].The formation of $[M-15]^+$, due to the loss of a methyl group from the TMS group, is another common fragment formed which allows for the inference of $[M]^{+\bullet}$. It was noted that $[M-15]^+$ for Ua and Ub, along with other fragment ions, were separated by a constant *m/z* difference of 28. Crucially, *m/z* 239.2359 ($[C_{16}H_{31}O]^+$; δ = −0.0010 Da) and 267.2675 ($[C_{18}H_{35}O]^+$; δ = −0.0007 Da) were indicative of a palmitate and stearate component in Ua and Ub respectively. Probable structures for Ua and Ub are, therefore, proposed as pentaerythritol monopalmitate (PMP), tris-TMS derivative and pentaerythritol monostearate (PMS), tris-TMS derivative respectively. Reference material was available for PMP to confirm its identity (Figure S4).

The structural assignment of Uc and Ud is more speculative. There were commonalities in characteristic HRMS data between Ua – Ud, with consistent detection of *m/z* 73.0455, *m/z* 147.0644, *m/z* 191.0904 and *m/z* 244.1284 (Figure S4). Furthermore, Uc had diagnostic fragment ions for a palmitate component, evident from *m/z* 239.2352 ($[C_{16}H_{31}O]^+$; δ = −0.0017 Da) and *m/z* 313.2547 ($[C_{18}H_{37}O_2Si]^+$; δ = −0.0010 Da), whereas Ud had diagnostic fragment ions for a stearate component, evident from *m/z* 267.2654 ($[C_{18}H_{35}O]^+$; δ = −0.0028 Da) and *m/z* 341.2854 ($[C_{20}H_{41}O_2Si]^+$; δ = −0.0016 Da). This suggested that Uc was a derivative of PMP, tris-TMS and Ud was a derivative of PMS, tris-TMS. Furthermore, $[M-15]^+$ showed a difference from PMP to Uc (assigned *m/z* 647.4202 ($[C_{32}H_{67}O_7Si_3]^+$; δ = 0.0013 Da)), of 72.0211 Da ($C_3H_4O_2$; δ = −0.0028 Da). This similarly applies to the difference between $[M-15]^+$ for PMS and Ud (*m/z* 675.4483 ($[C_{34}H_{71}O_7Si_3]^+$; δ = −0.0019 Da)). This indicated the inclusion of a lactic acid moiety for tentative structural assignment of Uc as PMP-[LA], tris-TMS derivative and Ud as PMS-[LA], tris-TMS derivative. The distinct detection of *m/z* 117.0710 ($[C_5H_{13}OSi]^+$; δ = −0.0020 Da) in the MS data of Uc and Ud supports this, as it is a common fragment ion of TMS functionalised secondary alcohols when bonded to a terminal methyl group (Figure S4).[9] In both instances, the specific structural isomer could not be determined. Both PMP-[LA], tris-TMS derivative and PMS-[LA], tris-TMS derivatives are presented in Figure S4 as the structural isomer of least steric hindrance but functionalisation at any OH terminal may be possible.

As additional confirmation, both PMP and PMS were detected through DI-Obitrap-MS and HPLC-Orbitrap-MS/MS as $[M+H]^+$ adducts which could be assigned accurate molecular formula and diagnostic fragment ions (*see Spreadsheet 7 –9*). PMS-[LA] and PMP-[LA] were assigned through HPLC-Orbitrap-MS/MS; albeit as $[M-H_2O+H]^+$ adducts in both cases as $[M+H]^+$ was below the intensity threshold for *dd*MS^2. The reference material for PMP was also prone to dehydration upon HESI ionisation to $[M-H_2O+H]^+$ which provided confidence in adduct assignment. PLA sequences extracted from the DI-Orbitrap-MS data indicated PMP-$[LA]_n$ derivatives up to n = 4 and PMS-$[LA]_n$ derivatives up to n = 6 (Table 1). Once again, specific structural isomers cannot be determined through DI-Orbitrap-MS data alone.

The source or function of the pentaerythtritol derivatives is ambiguous. Pentaerythritol has been recognised as a component of PLA/PBAT mulch film leachate elsewhere but not assigned a source.[11] The pentaerythritol mono-esters may be hydrolysis products of pentaerythritol fatty esters, which have a lubricating / slip agent function in the material.[12] To explain the formation of PMP-$[LA]_n$ and PMS-$[LA]_n$, hydrolysis reactions must have occurred between PLA components and hydroxyl groups of the pentaerythritol centre.[13] Another possibility is due to the incorporation of pentaerythritol as a branching agent during the polycondensation synthesis of PLA.[14] Incomplete polymerisation at this central molecule could account for the functionalisation with PLA oligomers of varying chain length. To form PMP-$[LA]_n$ and PMS-$[LA]_n$, however, this would require functionalisation of the pentaerythritol branching agent with fatty acid esters. Their source may also be due to the incorporation of pentaerythritol phosphites, such as disteary pentaerythritol diphosphite, which are used to facillitate the hydrolysis of PLA.[13] Polidar*, et al.* [13] notes the potential for lactic acid or PLA to react through hydroxyl or carboxyl groups of the phosphites. Subsequent hydrolysis for the removal of the phosphate from the pentaerythritol hydrolysis aid would result in the formation of similar pentaerythritol derivatives to those detected here.[15] Without proprietary information, a definitive assignment of the pentaerythritol chemical components source in the material cannot be made.

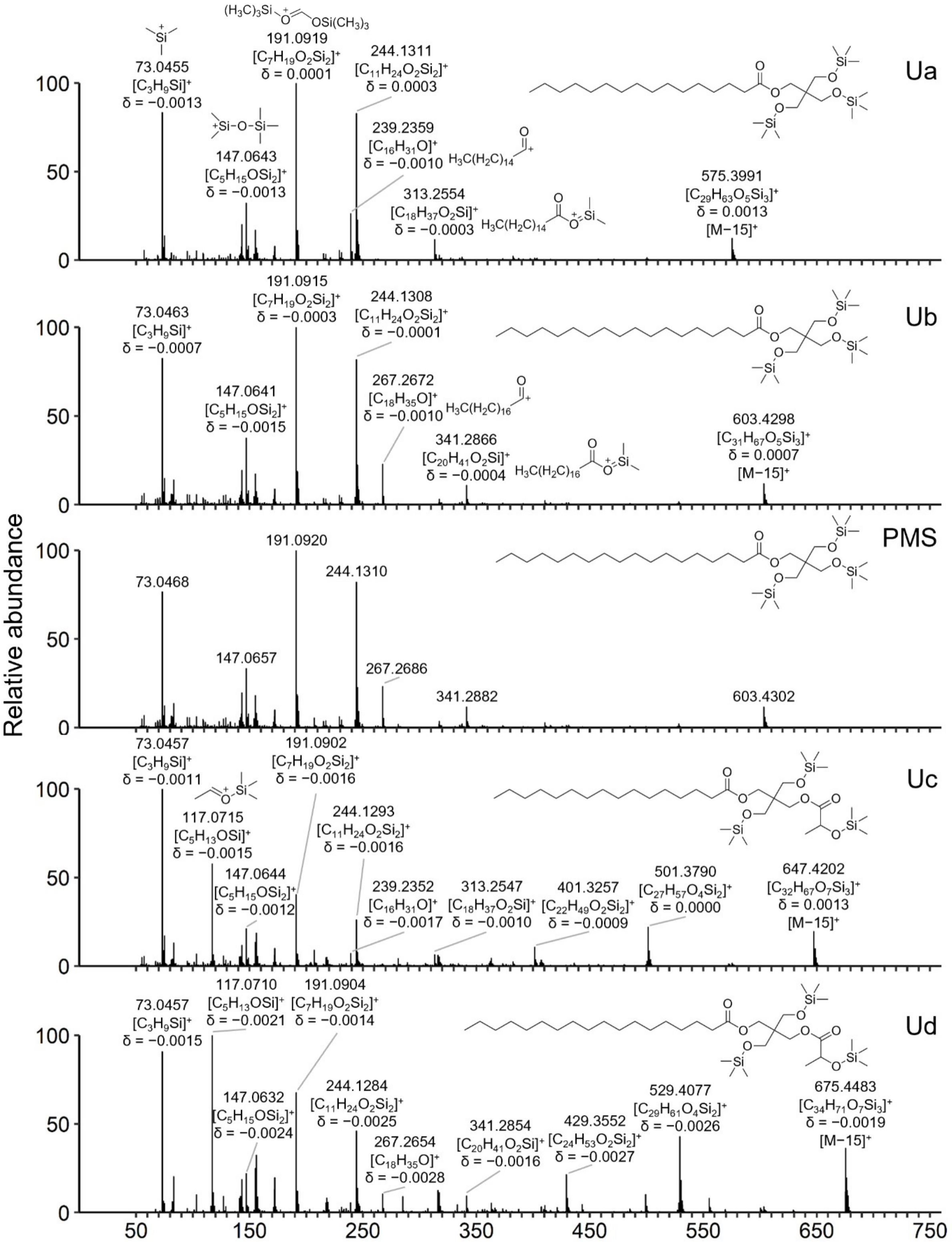

Figure S4: GC-Q-TOF-MS stacked spectra of Ua – Ud and PMS reference material (PMS), present in MeOH MSTFA derivatised extracts. Characteristic ions are labelled with assigned elemental formula and δ values in Da (difference between detected accurate mass of a given ion and the calculated exact mass for the assigned elemental formula of that ion).

S5. *DI-Orbitrap-MS – oligoester sequence extraction*

Initial data analysis was conducted using Xcalibur Version 4.1.31.9 (Thermo Fisher Scientific™ Ltd). HRMS data for each sample was averaged over 20 scans at 10% peak intensity of the TIC and exported as a separate Thermo .raw files containing only profile HRMS data. The .raw files were centroided and outputted as .mzML files using the MSConvertGUI software.[16] MS data handling for conversion to .csv files using the MSnbase package in R,[17,18] installed using the BiocManager package from the Bioconductor repository,[19] was undertaken for the extraction of *m*/*z* values, their corresponding intensities and RTs into a single data frame.

Oligomer signals, regardless of type (cyclic or linear) or the adduct formed, will exist in a sequence, separated by a constant *m*/*z* equivalent to the accurate mass of the monomeric unit. Therefore, to extract these ion signals from the data, all *m*/*z* values above an intensity threshold of 1E4 were inputted to a pairwise difference matrix within R. A list of expected mass differences of multiples (1 – 10) of the exact mass of the monomeric units for PLA (72.02113 Da), PBA (200.10486 Da), PBT (220.07356 Da) and PBSe (256.16746 Da) was then compared through a loop to the values in the difference matrix. If the absolute difference is a match to the expected difference within a mass tolerance of ± 0.001 Da, the corresponding *m*/*z* indices are populated into a separate list. Sequences of *m*/*z* indices are then constructed from this list, based on a constant mass difference of the monomer, building from an individual starting *m*/*z* in each case. Constructed sequences with less than 4 consecutive matches, including the starting *m*/*z* value, are then discarded to ensure robust identification of oligomer sequences. Finally, the sequence indices are converted to their actual *m*/*z* and intensity values from the source dataset to be output as a final sequence table in a .csv file for sorting, and elemental composition and identity assignment.

To ensure all extracted ions were oligomer components and PLA/PBAT mulch film derived, they had to be detected in all sample repeats (n = 3). Additionally, ions were removed if they were detected in any of the blank repeats (n = 3) within a tolerance of *m*/*z* ± 0.001 and at an average normalised intensity in sample repeats, normalised to the intensity of BBP, greater than 10% of the corresponding ion intensity in the blank.

S6. *PLA/PBAT Mulch Polymer Composition with ^{1}H NMR*

Table S2: Mulch film polymeric composition through ^{1}H NMR

Polymer	^{1}H NMR Signal[a]	*I* [b]	*m* [c]	Molar %	M_w repeat unit / g mol^{-1}	Molar wt. %
PBA	f (2.35 ppm)	3.50	4	35	128 + 72.1	35
PBT	a (8.12 ppm)	4.00	4	40	148 + 72.1	45
PLA	a' (5.19 ppm)	0.32	1	13	72.1	5
PBSe	E (1.32 ppm)	2.29	8	12	184 + 72.1	15

[a] ^{1}H NMR Signal is the representative ^{1}H nuclei environment for the repeat unit of each polymer.
[b] *I* is the intensity of the assigned resonance signal, proportional to the molar concentration of protons in signal a which is assigned a value of 4.
[c] *m* is the molar number of protons for the assigned resonance signal.

S7. *Chemical characterisation of dissolution-precipitation extracts of PLA/PBAT mulch film – GC-MS and GC-FID*

Table S3: Overview of organic compounds identified in solvent extracts of the PLA/PBAT mulch with GC-MS and quantification with GC-FID.

Assignment	RT range / min[b]	Match factor (probability) / %[c]	Characteristic ions (% abundance) / *m/z*	Confirmed with standard (✓)	RF[c]	Concentration[d] / $\mu g\ g^{-1}$
3,6-Dimethyl-1,4-dioxane-2,5-dione (lactide / cyclic $[LA]_2$)	10.18 - 10.19	82 – 86 (65 – 76)	56(100), 28(60), 43(37), 45(37), 55(11), 144(1)	✓		
3,6-Dimethyl-1,4-dioxane-2,5-dione (lactide / cyclic $[LA]_2$)	10.79 – 10.81	90 – 92 (57 – 60)	56(100), 28(60), 43(37), 45(37), 55(11), 144(1)	✓		
Cyclic [AA-BD] (1,6-dioxacyclododecane-7,12-dione / cyclic PBA monomer)	21.06	86 – 87 (93 – 95)	55(100), 54(73), 100(51), 41(40), 129(32), 111(31)	✓	0.60 ± 0.01	168 ± 32
Cyclic [SeA-BD] (1,6-dioxacyclohexadecane-7,16-dione / cyclic PBSe monomer)	29.90 – 29.91	-	55(100), 166(87), 98(70), 138(69), 54(62), 185(47)		*[AA-BD]*	588 ± 15
Tributyl aconitate (TBA)	33.92 - 33.93<br>34.17 – 34.18	92 – 93 (95 – 96)	112(100), 157(70), 57(55), 139(46), 41(44), 213(13)		*ATBC*	1400 ± 170
Tributyl citrate (TBC)	34.65 – 34.66	85 – 90 (82 – 86)	185(100), 129(98), 57(28), 41(24), 111(17), 259(16)		*ATBC*	104 ± 23
Acetyltributyl citrate (ATBC)	35.93 – 35.96	92 – 93 (96 – 97)	185(100), 129 (67), 259(44), 43(36), 57(22), 157(17), 139(13)	✓	0.67 ± 0.02	4200 ± 135
Cyclic $[AA\text{-}BD]_2$ (cyclic PBA dimer)	45.31	-	55(100), 129(89), 111(82), 127(46), 201(44), 54(44)	✓	0.59 ± 0.01	772 ± 5
Cyclic [AA-BD]-[TA-BD] (cyclic PBA-PBT monomers)	49.68	-	149(100), 104(71), 221(53), 55(49), 132(36), 111(28)		*$[TA\text{-}BD]_2$*	480 ± 12
Cyclic $[TA\text{-}BD]_2$ (cyclic PBT dimer)	53.02 – 53.03	-	132(100), 149(89), 54(62), 104(57), 121(39) 369(26)	✓	0.83 ± 0.04	348 ± 9

Table continues on next page

Table S2 *Continued*

Suspect	RT range / min[a]	Match factor (probability) / %[b]	Characteristic ions (% abundance) / *m/z*	Confirmed with standard (✓)	RF[c]	Concentration[d] / μg g^{-1}
Cyclic [TA-BD]-[SeA-BD] (cyclic PBT-PBSe monomers)	55.17 – 55.18	-	149(100), 104(54), 55(47), 221(44), 132(32), 166(31)		*[TA-BD]*$_2$	297 ± 7
Cyclic [SeA-BD]$_2$ (cyclic PBSe dimer)	55.85 – 55.86	-	55(100), 185(81), 71(43), 166(40), 127(40), 257(37)		*[AA-BD]*$_2$	252 ± 3
Unique suspects following MSTFA derivatisation						
Hexadecanoic acid, TMS derivative (palmitic acid, TMS derivative)	32.69	87 – 90 (95 – 96)	117(100), 313(52), 129(43), 132(43), 73(41), 145(33)	✓	-	4 ± 2
Octadecanoic acid, TMS derivative (stearic acid TMS derivative)	36.18	87 – 88 (94 – 98)	117(100), 129(51), 132(50), 341(47), 145(40), 73(36)	✓	-	18 ± 3
PMP, tris-TMS derivative	45.22	-	191(100), 244(76), 73(43), 147(43), 239(35), 143(24)			
PMS, tris-TMS derivative	47.70	-	191(100), 244(75), 147(39), 73(39), 267(24), 155(24)			
PMP-[LA], bis-TMS derivative	49.37	-	117(100), 191(36), 73(33), 156(23), 244(23), 147(22)			
PMS-[LA], bis-TMS derivative	51.62	-	117(100), 191(41), 73(33), 156(25), 244(24), 147(23)			

[a] Retention time (RT) range is presented as a range of elution times for the same compound between the triplicate solvent extracts.

[b] NIST library match factor is presented as a percentage range across repeats out of a total value of 1000 along with the probability range from the suspect list.

TMS – trimethyl silyl

[c] Response factors (RF) were taken as an average (n=3) across each GC-FID run to ensure consistency. Where compounds are listed, this informs the use of a surrogate standards RF for quantification relative to BBP. The RF of derivatised fatty acid decreased over the course of the GC run due to hydrolysis of the trimethylsilyl group or volatile losses respectively. As such, a RF value of 1 was used for quantification.

[d] Concentrations are expressed as the mean (n=3) ± 1 standard deviation.

References

1. Sipol®, Technipol® Bio 707, https://www.sipol.com/en/biodegradable-polymers-technipol-bio/technipol-bio-707/, (accessed 12th December 2022).
2. F. Khabbaz, S. Karlsson and A.-C. Albertsson, *Journal of Applied Polymer Science*, 2000, **78**, 2369-2378.
3. C. Westphal, C. Perrot and S. Karlsson, *Polymer Degradation and Stability*, 2001, **73**, 281-287.
4. A. Dhahak, C. Grimmer, A. Neumann, C. Rüger, M. Sklorz, T. Streibel, R. Zimmermann, G. Mauviel and V. Burkle-Vitzthum, *Waste Management*, 2020, **106**, 226-239.
5. F. De Falco, T. Nacci, L. Durndell, R. C. Thompson, I. Degano and F. Modugno, *Journal of Analytical and Applied Pyrolysis*, 2023, **171**, 105937.
6. C. Monkley, M. K. Reay, R. P. Evershed and C. E. M. Lloyd, *Rapid Communications in Mass Spectrometry*, 2024, **38**, e9726.
7. I. Coralli, A. G. Rombolà and D. Fabbri, *Journal of Analytical and Applied Pyrolysis*, 2024, **181**, 106577.
8. H. Cui, W. C. Gao, Y. C. Lin, J. Zhang, R. S. Yin, Z. M. Xiang, S. Zhang, S. P. Zhou, W. S. Chen and K. Cai, *Microchemical Journal*, 2021, **160**, 105722.
9. D. J. Harvey and P. Vouros, *Mass Spectrometry Reviews*, 2020, **39**, 105-211.
10. T. Curstedt, *Biochimica et Biophysica Acta (BBA) - Lipids and Lipid Metabolism*, 1974, **360**, 12-23.
11. H. Serrano-Ruiz, J. Eras, L. Martin-Closas and A. M. Pelacho, *Polymer Degradation and Stability*, 2020, **178**, 109202.
12. H. Zweifel, R. D. Maier and M. Schiller, *Plastic Additives Handbook*, Hanser Publishers, Munich, 6th edn., 2009.
13. M. Polidar, E. Metzsch-Zilligen and R. Pfaendner, *Polymers*, 2022, **14**, 4237.
14. L. Lopes Gomes Hastenreiter, S. K. Ramamoorthy, R. K. Srivastava, A. Yadav, A. Zamani and D. Åkesson, *Polymers*, 2020, **12**, 2849.
15. N. Ortuoste, N. S. Allen, M. Papanastasiou, A. McMahon, M. Edge, B. Johnson and K. Keck-Antoine, *Polymer Degradation and Stability*, 2006, **91**, 195-211.
16. M. C. Chambers, B. Maclean, R. Burke, D. Amodei, D. L. Ruderman, S. Neumann, L. Gatto, B. Fischer, B. Pratt, J. Egertson, K. Hoff, D. Kessner, N. Tasman, N. Shulman, B. Frewen, T. A. Baker, M.-Y. Brusniak, C. Paulse, D. Creasy, L. Flashner, K. Kani, C. Moulding, S. L. Seymour, L. M. Nuwaysir, B. Lefebvre, F. Kuhlmann, J. Roark, P. Rainer, S. Detlev, T. Hemenway, A. Huhmer, J. Langridge, B. Connolly, T. Chadick, K. Holly, J. Eckels, E. W. Deutsch, R. L. Moritz, J. E. Katz, D. B. Agus, M. MacCoss, D. L. Tabb and P. Mallick, *Nature Biotechnology*, 2012, **30**, 918-920.
17. L. Gatto, S. Gibb and J. Rainer, *Journal of Proteome Research*, 2021, **20**, 1063-1069.
18. R Core Team, 2022. *R: A Language and Environment for Statistical Computing*; R Foundation for Statistical Computing: Vienna, Austria. https://www.R-project.org/.
19. M. Morgan and M. Ramos, 2024. *BiocManager: Access the Bioconductor Project Package Repository*; https://CRAN.R-project.org/package=BiocManager.
